# NR5A2 (located on chromosome 1q32) inhibits ferroptosis and promotes drug resistance by regulating phospholipid remodeling in multiple myeloma

**DOI:** 10.7150/ijbs.113115

**Published:** 2025-09-08

**Authors:** Panpan Li, Jiadai Xu, Bei Xu, Xiaowen Hu, Yaqin Xiong, Yawen Wang, Peng Liu

**Affiliations:** 1 Department of Hematology, Zhongshan Hospital, Fudan University, Shanghai, China.; 2 Cancer Center, Zhongshan Hospital, Fudan University, Shanghai, China.; 3 Department of Hematology, Zhongshan Hospital (Xiamen), Fudan University, Xiamen, Fujian, China.; 4 Department of Medical Oncology, Zhongshan Hospital, Fudan University, Shanghai, China.; 5 Bio-X Institutes, Key Laboratory for the Genetics of Developmental and Neuropsychiatric Disorders (Ministry of Education), Shanghai Jiao Tong University, Shanghai 200030, China.

**Keywords:** Multiple myeloma, NR5A2, MBOAT1, MBOAT2, phospholipid remodeling, ferroptosis

## Abstract

Multiple myeloma (MM) is a prevalent hematologic malignancy with improved survival rates over recent decades, although still uncurable. MM with chromosome 1q Gain (1q+) are clinically and biologically heterogeneous. In this study, we found that NR5A2, located on chromosome 1q and encoding an essential transcriptional regulator of lipid metabolism, has higher mRNA expression in 1q+ patients and could further stratify the prognosis of MM patients. Omics data were analyzed and related experiments were conducted. We demonstrated for the first time that NR5A2 promotes the proliferation and invasion of MM cells by regulating phospholipid metabolism and further inhibit ferroptosis by reducing the related specific substrate in MM cells. Through integrated analysis of the lipid metabolism and proteome, MBOAT1 and MBOAT2 were determined to be the downstream targets of NR5A2. Furthermore, it has been determined that the high expression of NR5A2 is closely related to the resistance of MM cells to dexamethasone (Dexa). Interestingly, we found for the first time that arachidonic acid co-culture with MM cells can promote their sensitivity to Dexa and significantly reverse the resistance to Dexa caused by high expression of NR5A2. These findings provide insights into disease-causing mechanisms and new therapeutic targets for MM patients with 1q+.

## Introduction

Multiple myeloma (MM), presenting with abnormal proliferation of plasma cells, is the second most common hematologic malignancy after non-Hodgkin's lymphoma^1^,^2^. The survival of MM patients has been significantly improved with the advent of novel therapeutic approaches in recent decades. However, MM is still uncurable^2^-^4^ Chromosome 1q Gain (1q+) is the most common cytogenetic abnormality in MM, with a detection rate of 30-50% and 70% in individuals with newly diagnosed MM (NDMM), and those with relapsed refractory MM (RRMM), respectively^5^. Furthermore, there may be higher likelihood of relapse in MM patients with 1q+^6^.

In the context of increased metabolic demands of highly proliferative tumor cells, it promotes the reprogramming of various metabolic pathways, including fatty acid oxidation, mitochondrial energy metabolism, etc^7^. Recent studies have elaborated the relationship between MM and lipid metabolism^8^,^9^. For example, the overall survival (OS) is significantly lower in obese MM patients under the age of 65 than that of the normal group^10^, while patients with higher levels of apolipoprotein A1 exhibit longer OS^11^. In addition, Noopur Raje et al. discovered promoted proliferation of MM cells under low concentrations of free fatty acids^12^.

Lipid metabolism plays a central role in cellular ferroptosis, a form of programmed cell death that is propelled by iron-dependent peroxidation of phospholipids (PLs), with plasma membrane rupture as its ultimate manifestation^13^. PLs undergo endogenous synthesis and dynamic remodeling through fatty acyl deacylation and reacylation, a process referred to as the land's cycle. It comprises two steps^14^: the catalysis of PL deacylation by phospholipase A2, and the mediation of lysophospholipid reacylation by lysophosphatidylcholine acyltransferase (LPCAT). These two enzymes are potential regulators of ferroptosis. Currently, we know little about the specific relationship between lipid metabolism, ferroptosis, and drug resistance in MM cells.

The protein encoded by NR5A2 serves as a crucial transcriptional regulator in lipid metabolism^15^. In 2005, the team led by Holly A. Ingraham indicated that PLs can serve as ligands to directly bind to NR5A2. In addition, high expression of NR5A2 has been reported to be closely related to tumor drug resistance. In T-cell leukemia and prostate cancer, NR5A2 exhibited resistance to glucocorticoid^16^ and darolutamide^17^ respectively. However, so far, there is currently no report on the relationship between NR5A2 and drug resistance in MM cells.

Divertingly, MBOAT1/2 have been reported to enable an independent inhibition of ferroptosis directly, apart from glutathione peroxidase 4 (GPX4, a key regulator of ferroptosis)^18^. However, there have been no reports on the relationship of NR5A2 with the MBOAT superfamily and ferroptosis. Accordingly, in this study, we explored the expression of NR5A2 in 1q+ MM patients, and its association with poor prognosis in these patients. Then, this study continued to investigate the relationship of NR5A2 with MBOAT1 and MBOAT2 (downstream effectors) through analyses omics and proteomics and CUT&Tag qPCR. Furthermore, we sought to investigate the internal mechanisms by which the NR5A2-MBOAT1/2 axis promoted MM cell proliferation and invasion. The findings of this study may offer potential new targets for the treatment of MM.

## Methods

### Patients and samples

In this study, 27 NDMM and 22 healthy donors (HD) were recruited for niacin flush test Table S1. Detailed methods are described in the Supplementary methods. For whole exon sequencing (WES), bone marrow mononuclear cells (BMMC)^19^ collected from 7 monoclonal gammopathy of undetermined significance (MGUS) patients, 5 smoldering MM (SMM), 47 NDMM and 17 RRMM patients were included in this study Table S2. From 2013 to 2021, 584 NDMM who underwent ferritin and FISH (Fluorescence *in situ* Hybridization) were contained in this study. The electronic records of the relevant cases were examined, and comprehensive clinical data are provided in the Table S3.

The CoMMpass study, registered as a prospective observational clinical trial (NCT01454297), entails thorough genomic and transcriptomic profiling of individuals diagnosed with NDMM. Data acquisition was facilitated through the MMRF Researcher Gateway (https://research.themmrf.org).

The diagnoses of MGUS, SMM, MM, as well as the definition of progressive disease (PD), were established following the criteria in accordance with the International Myeloma Working Group (IMWG) in 2018^20^ 2010^21^, and 2016^22^.

Under the terms of informed permission, this study adhered to the World Medical Association's Declaration of Helsinki and was approved by the Ethics Committee (B2017-031R) of Zhongshan Hospital, Fudan University.

### Description of the experimental Method

The adenosine triphosphate (ATP) content was assessed using an Enhanced ATP Assay Kit (Beyotime, S0027) following the manufacturer's instructions. The intracellular levels of reactive oxygen species (ROS) in HMCLs were quantified utilizing a Reactive Oxygen Species Assay Kit with the DCFH-DA probe (Beyotime, S0033M). The cellular oxygen consumption rates (OCRs) were assessed using the Seahorse XF Cell Mito Stress Test Kit (Seahorse Bioscience, USA) with an XFe96 Extracellular Flux analyzer. The intracellular levels of Fe^2+^ in HMCLs were quantified utilizing a Fe2+ indicator (Shanghai Maokang BiotechnologyCo). CUT-Tag was performed according to the instructions^23^. Quantitative real-time polymerase chain reaction (qRT-PCR) Table S4 and Western blot (WB) Table S5, proteome profiling and comprehensive metabolomics-lipidomics, CCK-8 assay, trans-well assay, transmission electron microscopy (TEM), Malondialdehyde (MDA) and Oil red O staining were performed.

## Results

### High expression of NR5A2 in 1q+ MM patients and its predictive role of poor prognosis in MM patients

**Figure 1:**
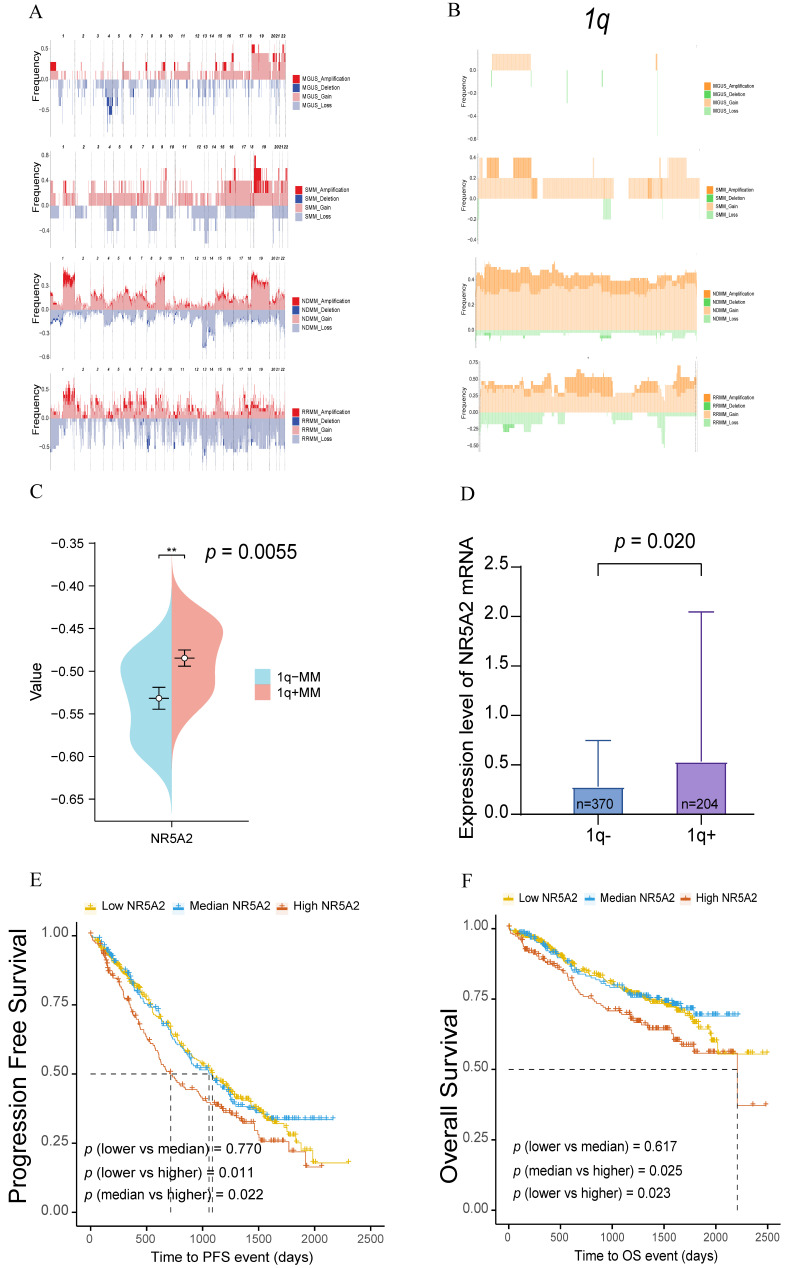
A. Comparison of the ratio of chromosomal gene amplifications and deletions in 47 NDMM, 5 SMM, 7 MGUS, and 17 RRMM in our center. Genomic landscape of chromosomal amplifications/deletions in NDMM (n=47), SMM (n=5), MGUS (n=7), and RRMM (n=17) patients. B. Ratio of chromosomal 1q segment gene amplification and deletion across MM disease stages. C. NR5A2 exhibited high mRNA expression in patients with 1q+(p = 0.0055). D. NR5A2 exhibited high mRNA expression in patients with 1q+ in MMRF Compass (p = 0.020). E. Elevated NR5A2 mRNA expression correlated with inferior progression-free survival (p = 0.011) in multiple myeloma patients from the MMRF CoMMpass cohort. F.High NR5A2 mRNA expression was associated with worse OS in the MMRF CoMMpass MM cohort (p = 0.023).

According to an analysis of WES data from 47 NDMM, 5 SMM, 7 MGUS, and 17 RRMM patients, high frequency of gene amplifications or deletions were found during the clonal evolution of MM compared to MGUS and SMM, especially for chromosome 1 abnormalities (Figure 1A). Further analysis of the chromosome 1q region revealed more frequent gene amplifications or deletions in NDMM and RRMM patients than in those with MGUS and SMM (Figure 1B), highlighting the critical role of 1q in MM tumor progression.

Based on further analysis of the WES data from our center, 20 transcription factors with significantly amplified gene copy numbers were identified in NDMM patients compared to those with MGUS. The specific markers involved NHLH1, GON4L, USF1, PRRX1, NR5A2, PIAS3, ETV3, NR1I3, ATF6, TBX19, BLZF1, ZBTB37, LHX4, ZNF648, ZBTB41, LHX9, ZNF124, ZNF496, ZNF672, and ZNF692 Supplement Figure 1A). Through subsequent validation using RNA-Seq data from 29 MM patients in another cohort of our center, amplification of the NR5A2 gene, located in the 1q32 region, led to elevated transcriptomic expression (Figure 1C, *p*=0.006; Supplement Figure 1B). This study continued to examine the expression of transcription factors in 1q- and 1q+ MM patients and their mRNA levels in relation to survival outcomes, as supported by data from the MMRF CoMMpass database, including FISH data from 574 MM patients, with elevated NR5A2 expression detected in 1q+ MM patients (Figure 1D, *p*=0.020). In additional, based on the survival data of these 574 MM patients, compared to those with low expression, MM patients with high expression of NR5A2 showed significantly worse progression-free survival (PFS, *p*=0.011) and OS (*p*=0.023) (Figure 1E, 1F).

### Promoting roles of NR5A2 in the proliferation and invasion of human myeloma cell lines (HMCLs)

**Figure 2:**
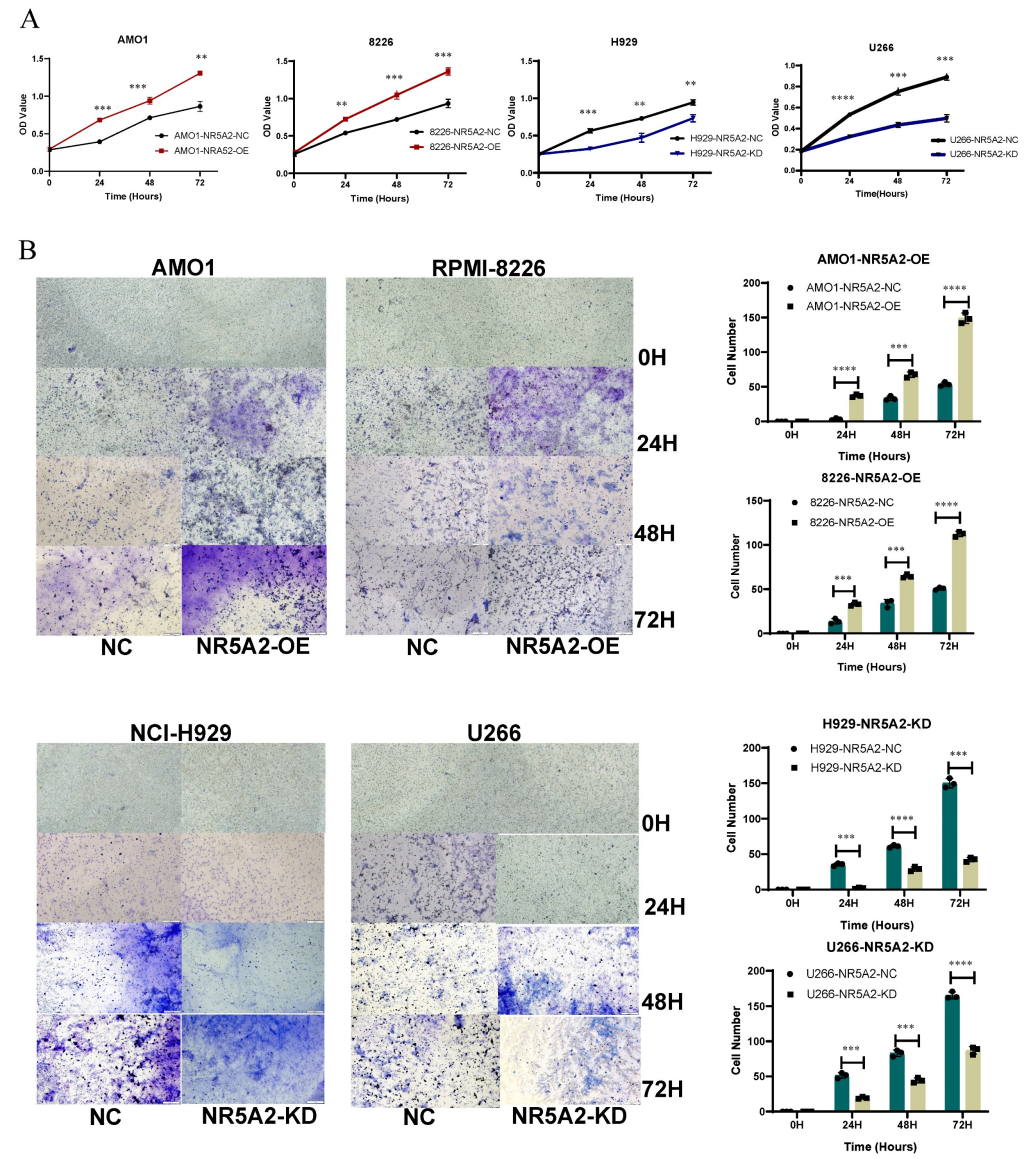
A. CCK-8 detection experiments confirmed that high expression of NR5A2 can promote proliferation of HMCLs. B. Tans-well invasion experiments indicated that NR5A2 promotes invasion of HMCLs.

We validated the expression of NR5A2 at mRNA and protein levels in HMCLs, respectively. The expression of NR5A2 was low in AMO1 and RPMI-8226 cells, but high in NCI-H929 and U266 cells (Supplement Figure 2A), respectively. Accordingly, this study established a stable NR5A2-overexpressing (OE) in AMO1 and RPMI-8226 cells, and NR5A2-knockdown (KD) in H929 and U266 cells (Supplement Figures 2B and 2C). As evidenced by the CCK-8 assay to evaluate the impact of NR5A2 on MM cell proliferation, the OE of NR5A2 stimulated MM cell proliferation (Figure 2 A). Furthermore, Trans-well assay hinted significantly enhanced invasion in AMO1-NR5A2-OE and RPMI-8226-NR5A2-OE groups, while declined invasion in NCI-H929-NR5A2-KD and U266-NR5A2-KD groups (Figure 2 B). In addition, we conducted an exploration in the OCI-MY5 (1q-), and the results revealed that OCI-MY5-NR5A2-OE had a statistically significant difference compared with the control only at 72 hours (*p*=0.0009). The trans-well invasion assay showed that OCI-My5-NR5A2-OE had statistically significant differences compared with the NC group at 48 (*p*=0.018) and 72 hours (*p*=0.005) (Supplement Figure 3. Compared with the significant difference in 1q+ MM cells as shown in Figure 2 and the significantly higher expression of NR5A2 in 1q+ MM patients as shown in Figure 1, these pieces of evidence suggest that NR5A2, is of great significance to 1q+ MM patients.

### NR5A2 inhibits ferroptosis in HMCLs

**Figure 3:**
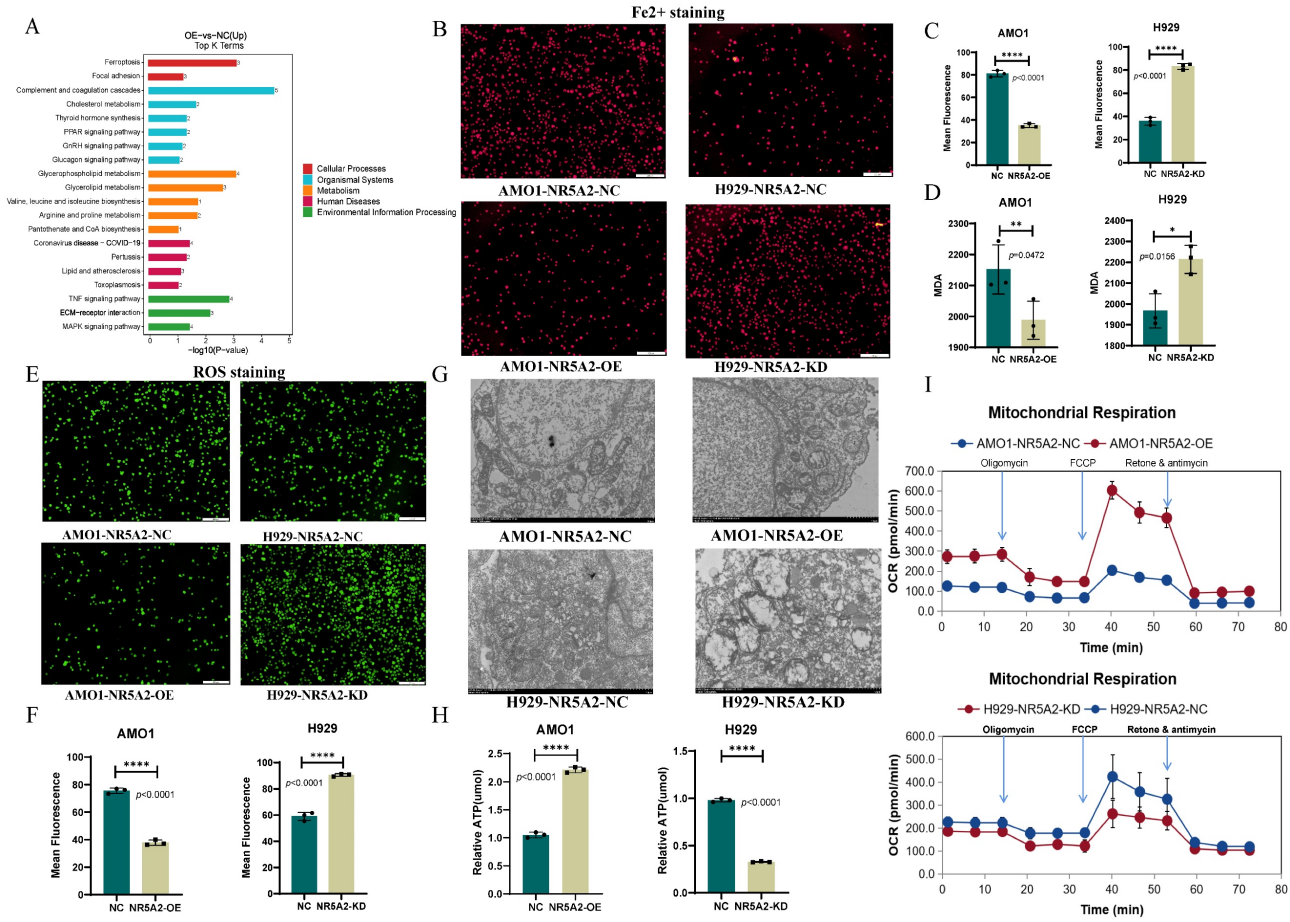
A. KEGG enrichment analysis of NR5A2 OE and NR5A2 NC. B-C. Fe2+ staining showed that after NR5A2 OE, the fluorescence intensity was weakened. D. Decreased malondialdehyde (MDA) in NR5A2-OE cells. E-F. ROS staining showed that after NR5A2 OE, the fluorescence intensity was weakened. G. After NR5A2 KD, transmission electron microscopy reveals fractionated mitochondrial cristae structure. H. After NR5A2 OE, there is an increase in ATP production. I. After NR5A2 OE, the OCR significantly increases.

KEGG pathway enrichment analysis on proteomics data between AMO1-NR5A2-NC and AMO1-NR5A2-OE revealed the involvement of NR5A2 in ferroptosis pathways and glycerolphospholipid metabolism (Figure 3 A). Given that Fe^2+^ is a substrate for ferroptosis, Fe^2+^ staining was conducted to further elucidate whether NR5A2 affected ferroptosis in HMCLs. AMO1-NR5A2-OE group had weaker red fluorescence than that of the AMO1-NR5A2-NC group (*p*<0.001), indicating that NR5A2 regulates Fe²⁺ levels (Figure 3B, 3C). Furthermore, MDA is a key product of membrane lipid peroxidation, also serving as an indicator of this process. MDA levels in the AMO1-NR5A2-OE group were significantly lower than those in the AMO1-NR5A2-NC group (*p*<0.001) (Figure 3D). In addition, ferroptosis is often accompanied by an increase in ROS accumulation. Hence, with the measurement of ROS, weaker green fluorescence was detected in the AMO1-NR5A2-OE group than that in the NR5A2-NC group (*p*<0.001) (Figure 3E, 3F).

The effect of NR5A2 on mitochondrial structure and function was also investigated to unveil the regulation of NR5A2 on HMCLs ferroptosis. TEM showed that the KD of NR5A2 led to more fractured mitochondrial cristae structures in HMCLs (Figure 3G). Then ATP level was measured to be significantly lower in NCI-H929-NR5A2-KD cells than NC group (*p*<0.001) (Figure 3H). Finally, compared to the AMO1-NR5A2-NC group, the AMO1-NR5A2-OE group exhibited significant increases in OCRs (Figure 3I; Supplement Figure 4A).

In further analysis on the clinical data from 584 MM patients with FISH results at our center, compared to 1q- MM patients, 1q+ MM patients exhibited significantly increased ferritin levels (*p*=0.009), obviously decreased levels of high-density lipoprotein cholesterol (*p*=0.003), apolipoprotein A1 (*p*=0.001), and apolipoprotein (a) (*p*=0.030) Supplement Figure 4 B). Therefore, patients with 1q+ MM were observed with suppressed ferroptosis and dysregulated lipid metabolism, which were consist with our *in vitro* experiments.

### NR5A2 inhibits ferroptosis in HMCLs by regulating phospholipid remodeling

**Figure 4:**
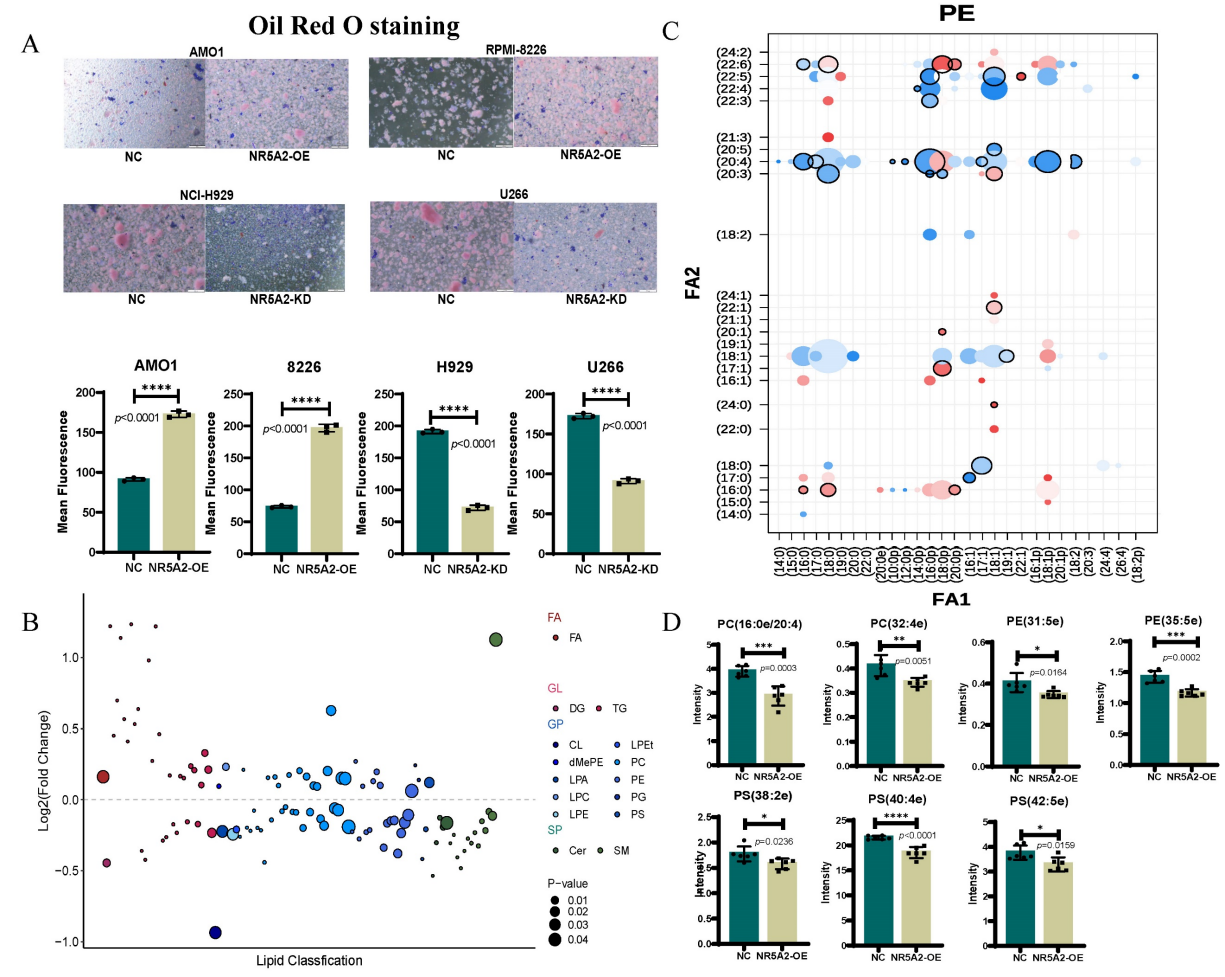
A. Oil Red O staining showing lipid accumulation in NR5A2-OE HMCLs. B. After overexpression of NR5A2, the most significant change in lipids is observed in glycerophospholipids. C. The changes of various lipids in phosphatidylethanolamine (PE) after NR5A2 overexpression. D. After overexpression of NR5A2, the expression abundance of PUFA containing ether lipids were significantly decreased.

To further validate the impact of NR5A2 expression on lipid metabolism in HMCLs, Oil Red O staining was performed. The AMO1-NR5A2-OE group had a significant increase in lipid content compared to the AMO1-NR5A2-NC group (*p*<0.001) (Figure 4A). Subsequently, untargeted lipid metabolomics revealed significant alterations in the AMO1-NR5A2-OE group compared to the AMO1-NR5A2-NC group (Figure 4B).

Previous studies have shown that membrane PLs containing polyunsaturated fatty acids (PUFA) are critical substrates for ferroptosis(13, supporting our exploration of changes in fatty acids in PLs after NR5A2 overexpression. Phosphatidylethanolamine (PE) with a single arachidonic acid (AA) or 22:4 PUFA tail, PLs containing two PUFA tails, or PUFA-containing ether lipids have been identified as specific lipids driving ferroptosis^24^. In our study, after NR5A2 overexpression, there were decreased. PE containing one AA or one Adrenaline Acid (AdrA) (Figure 4C), and significantly reduced expression abundances of ether lipids containing PUFA (Figure 4 D). Collectively, NR5A2 overexpression resulted in obviously decreased expression abundances of most specific lipids driving ferroptosis, further confirming the inhibition of ferroptosis in HMCLs after NR5A2 overexpression.

Niacin-induced skin flushing was considered as a marker of the levels of AA and AdrA^25^,^26^. Skin niacin flush response tests were conducted on 27 MM patients and 22 HD collected at our center. The clinical baseline characteristics are presented in Table S1. MM patients exhibited a significantly attenuated niacin flushing response compared to healthy controls (*p* = 0.0005). Patients with 1q+ MM demonstrated a significantly reduced niacin flushing response relative to those with 1q- MM (*p* = 0.0439), indicating a deficiency of AA and AdrA in MM patients. No significant change was observed in the niacin flushing reactivity of MM patients before versus after treatment. Among 16 longitudinally followed MM patients, those with high niacin flushing reactivity showed longer OS and PFS than patients with low reactivity. Compared to HD, MM patients exhibited a significantly decreased niacin flush response Supplement Figure 5.

### NR5A2 regulates MBOAT1/2 to inhibit non-GPX4-dependent ferroptosis in HMCLs

**Figure 5:**
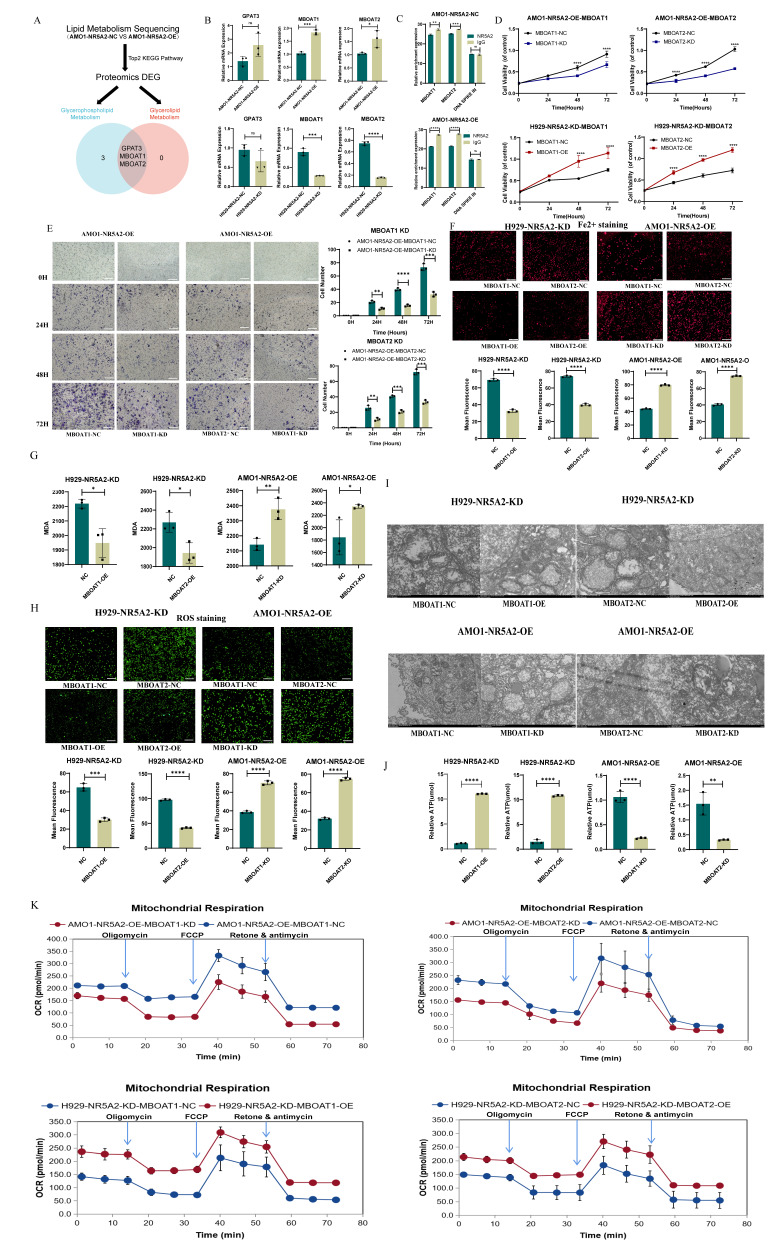
A. Key Downstream Molecule Screening Flowchart. B. PCR confirmed that MBOAT1 and MBOAT2 exhibit concordant changes with NR5A2 at the mRNA level. C. CUT-Tag validation demonstrated that NR5A2 can regulate the expression of MBOAT1 and MBOAT2. D. CCK-8 assay reveals that NR5A2 affects HMCLs proliferation by regulating the expression of MBOAT1 and MBOAT2. E. Trans-well assay reveals that NR5A2 affects HMCLs invasion by regulating the expression of MBOAT1 and MBOAT2. F. NR5A2 affected the content of Fe2+ in HMCLs through MBOAT1 and MBOAT2. G. NR5A2 affected the generation of MDA in HMCLs through MBOAT1 and MBOAT2. H. NR5A2 affected the generation of ROS in HMCLs through MBOAT1 and MBOAT2. I. TEM observation revealed that NR5A2 affects mitochondrial cristae structure via MBOAT1 and MBOAT2. J. NR5A2 affected the generation of ATP in HMCLs through MBOAT1 and MBOAT2. K. NR5A2 affected mitochondrial energy metabolism in HMCLs through MBOAT1 and MBOAT2.

To further explore the downstream regulatory network of NR5A2, we performed a comprehensive screening in lipidomics and proteomics comparing NR5A2-OE and NR5A2-NC, as illustrated in Figure 5A. This analysis revealed GPAT3, MBOAT1, and MBOAT2 as potential downstream targets.

Subsequent validation of the mRNA levels of these molecules showed that MBOAT1 and MBOAT1 changed in the same direction as NR5A2 at the mRNA level (Figure 5B). CUT-Tag qPCR to validate the relationship between NR5A2 and MBOAT1/MBOAT2 indicated that NR5A2 could regulate the expression of MBOAT1 and MBOAT2 (Figure 5C).

Further investigation focused on the exploration of whether NR5A2 affects the proliferation, invasion and ferroptosis of HMCLs through MBOAT1 and MBOAT2. Firstly, overexpression and knockdown experiments were conducted for MBOAT1 and MBOAT2 in H929-NR5A2-KD and AMO1-NR5A2-OE cells to perform phenotype restoration, with the verification of the mRNA and protein levels Supplement Figure 6A; Supplement Figure 6B). Meanwhile, MBOAT1 and MBOAT2 could reverse the effect of NR5A2 on HMCLs cell proliferation and invasion, as indicated by the results of CCK-8 and Trans-well assays (Figure 5D; Figure 5E). Subsequently, according to a series of well-established ferroptosis assays, the H929-NR5A2-KD ^MBOAT1/2-OE^ group was detected with significantly decreased Fe²⁺ and MDA (Figure 5 F; Figure 5G*, p*<0.001), as well as reduced ROS level (Figure 5H,* p*<0.001). TEM observation revealed that the H929-NR5A2-KD^MBOAT1/2-OE^ cells had clearer inner mitochondrial cristae structures (Figure 5I). The H929-NR5A2-KD^MBOAT1/2-OE^ group was also observed with remarkably increased ATP level (*p*<0.001) (Figure 5J). In rescue OCRs, the AMO1-NR5A2-OE^MBOAT1/2-OE^ group had obviously declined spare respiratory capacity and non-mitochondrial oxygen consumption when compared to the AMO1-NR5A2-OE^MBOAT1/2-NC^ group (Figure 5K; Supplement Figure 7A). These findings, supported by strong statistical results, indicated the regulatory role of NR5A2 in ferroptosis by MBOAT1 and MBOAT2.

To further elucidate the mechanism by which NR5A2 inhibits ferroptosis in MM cells, we first examined the expression of key ferroptosis regulators(27,28: GPX4, SLC7A11, and ACSL4 in cell lines. As shown in Supplementary Figures 8A and 8B, NR5A2 modulation had no significant effect on GPX4 or SLC7A11 expression at either the mRNA or protein level.

Intriguingly, we observed that overexpression of NR5A2 markedly downregulated ACSL4, a critical pro- ferroptosis regulator. ACSL4 catalyzes the activation of long-chain PUFA, particularly AA and adrenic acid (AdA), into their respective acyl-CoA esters (AA-CoA, AdA-CoA)^29^. This process increases the pool of specific substrates susceptible to peroxidation during ferroptosis, aligning with our lipid metabolism findings. Furthermore, immunohistochemical analysis of murine xenograft tumors corroborated this conclusion Supplement Figure 8 C).

### Arachidonic acid reverses dexamethasone resistance in NR5A2-overexpressing HMCLs

**Figure 6:**
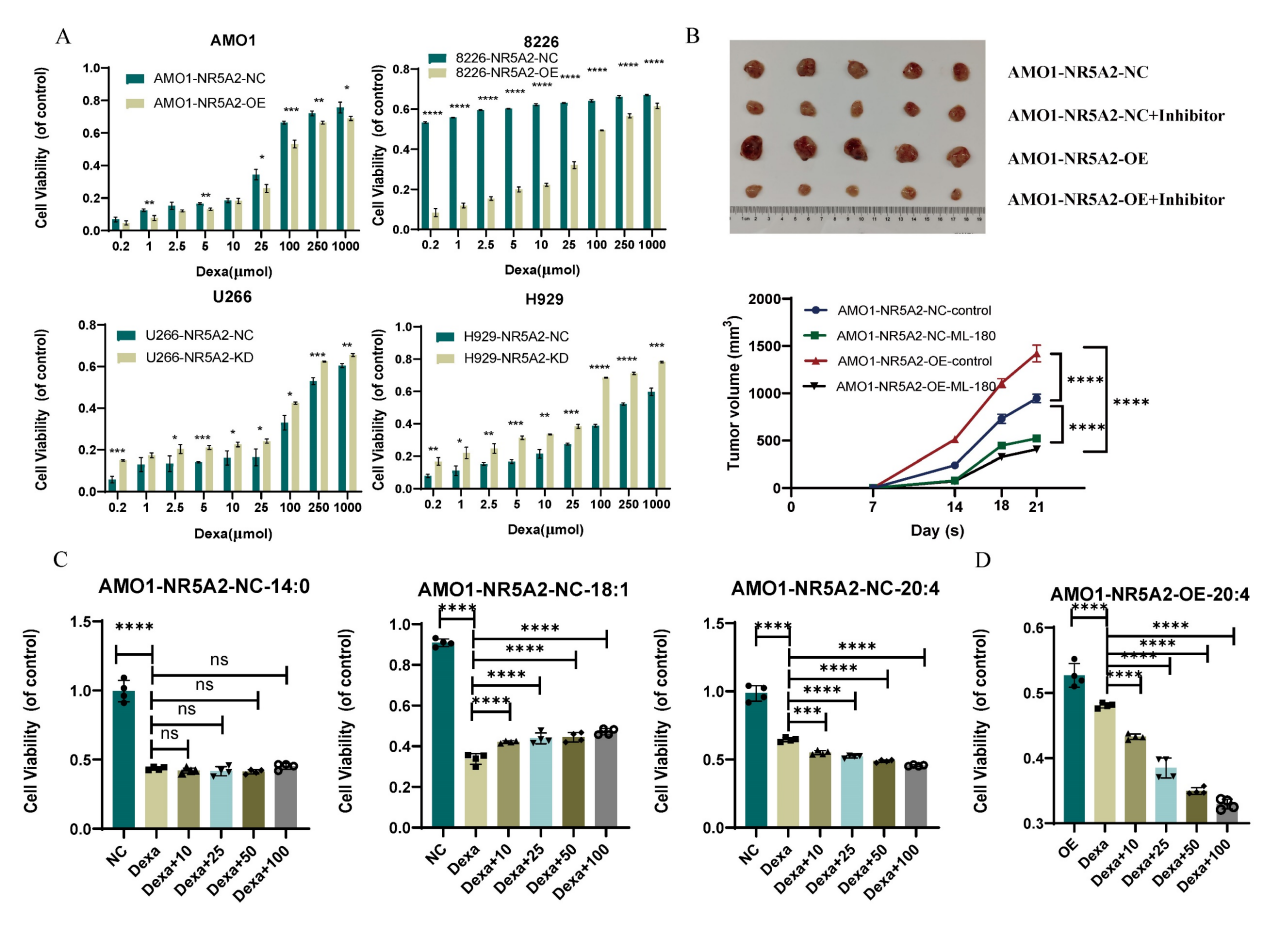
A. NR5A2-OE confers dexamethasone resistance, while NR5A2-KD sensitizes HMCLs to dexamethasone. B. Xenograft tumor experiments in mice suggested that NR5A2 can promote proliferation of HMCLs *in vivo* and NR5A2 inhibitor ML-180 significantly inhibited tumor growth in mice upon administration. C. Proliferation assay of fatty acid co-culture with AMO1-NR5A2-NC, including SFA (14:0), MUFA (18:1), and PUFA (20:4). D. Proliferation assay of PUFA (20:4) co-culture with AMO1-NR5A2-OE.

To validate the effect of the NR5A2 inhibitor ML-180 on MM proliferation *in vivo*, we conducted xenograft tumor experiments in NOG-SCID mice. Results revealed significant increase in tumor volume in the AMO1-NR5A2-OE group compared to that in the NR5A2-NC group. Moreover, ML-180 significantly inhibited tumor growth, with a stronger effect in the AMO1-NR5A2-OE group (Figure 6B), indicating that MM patients might benefit more from NR5A2 inhibition.

Previous studies have shown competitive binding of NR5A2 to the glucocorticoid receptor to trigger glucocorticoid resistance (16. Accordingly, this study found promoted Dexa sensitivity in the NR5A2- KD group (Figure 6A). Additionally, under treatment with 100 μmol Dexa, the levels of ROS, Fe²⁺, and MDA were reduced in the NR5A2-OE group, but increased in the NR5A2-KD group Supplement Figure 9 A-C).

A detailed analysis of lipid metabolism data revealed significant changes in myristic acid (MA, 14:0), oleic acid (OA, 18:1), and AA within saturated (SFA), monounsaturated (MUFA), and PUFA (Supplement Figure 10. Co-culture experiments were employed to assess whether these fatty acids affected HMCL sensitivity to Dexa. Consequently, MA did not affect, whereas OA decreased, and AA increased Dexa sensitivity (Figure 6C). We also verified that AA could reverse Dexa resistance induced by high NR5A2 expression in MM cells (Figure 6D).

Finally, upon literature review, XPO1 emerges as a pivotal nuclear export protein. Selective XPO1 inhibitors (e.g., Selinexor) can bind to XPO1, leading to the activation of anti-cancer proteins and restoration of hormone sensitivity. NR5A2 can compete with the glucocorticoid receptor, resulting in glucocorticoid resistance^16^. Based on this, we conducted assays to determine if Selinexor can counteract Dexa resistance induced by high NR5A2 expression. Our experiments showed that Selinexor significantly inhibited NR5A2 expression Supplement Figure 11 A) and counteracted Dexa resistance (Supplement Figure 11 B).

## Discussion

**Figure 7:**
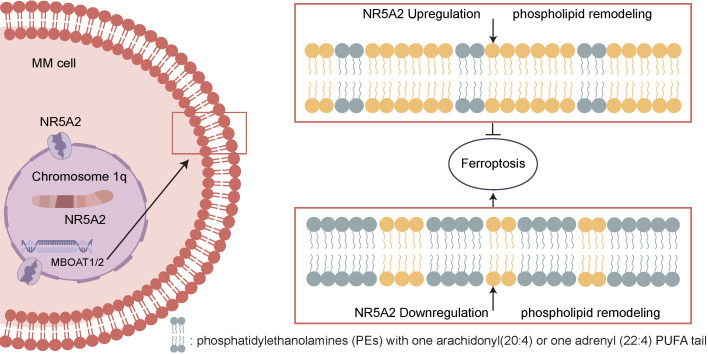
NR5A2-MBOATI/MBOAT2 axis inhibits GPX4-independent ferroptosis of MM cells by regulating phospholipid remodeling and promotes the malignant proliferation of MM cells.

MM patients with 1q+ usually have drug resistance and poor prognosis, remaining a significant challenge to be adequately addressed. Identifying the mechanisms driving the malignant phenotype from clinical samples can help pinpoint precise therapeutic targets for clinical translation. In this study, we identified NR5A2 as a key transcription factor in a large cohort of 1q+ MM patients using WES. The NR5A2-MBOAT1/MBOAT2 axis can inhibit ferroptosis in tumor cells, promoting tumor progression. Moreover, for the first time, this study proposes that NR5A2 inhibitors can reverse Dexa resistance, offering a novel therapeutic strategy (Figure 7.

Copy number variations are often associated with genomic instability and malignant tumor phenotypes^30^,^31^. This study intended to decipher the unique oncogenic mechanisms in 1q+ MM patients by comprehensively analyzing genes in the 1q region using WES data from our center, combined with the CoMMpass database. Strikingly, NR5A2, located in the 1q32 region, was significantly overexpressed in 1q+ patients compared to 1q- patients; and elevated NR5A2 expression was correlated with shorter PFS and OS in a large patient cohort. Consistently, previous studies have linked NR5A2 to malignant phenotypes in pancreatic cancer^32^, glioma^33^, and cutaneous squamous cell carcinoma^34^, etc. Further *in vitro* and *in vivo* experiments confirmed the pivotal oncogenic role of NR5A2 in 1q+ MM, which may be a key molecular marker in this subgroup of patients.

Given the crucial role of NR5A2 in 1q+MM patients, this study continued to explain its mechanisms by employing proteomics and non-targeted lipid metabolomics. With the integration of sequencing results, NR5A2 was found to have an intimate association with ferroptosis in MM patients (Figure 3A), coupled with validation through a series of experiments. Ferroptosis, a form of programmed cell death characterized by iron-dependent lipid peroxidation^35^,^36^, has been shown in prior studies to involve key regulators such as GPX4, SLC7A11 and ACLS4^37^. This study demonstrated that NR5A2 suppresses ferroptosis in MM cells primarily by downregulating the expression of ACSL4, a critical pro- ferroptosis regulator. The key function of ACSL4^29^,^38^ is to promote the biosynthesis of pro- ferroptosis phospholipid polyunsaturated fatty acids (PUFA-PLs). These PUFA-PLs serve as essential substrates for lipid peroxidation during the execution phase of ferroptosis. This mechanism aligns precisely with our lipidomic findings. Through integrative omics analyses and CUT-Tag qPCR, MBOAT1/MBOAT2 were identified as downstream mediators of NR5A2. Liang et al.^39^ have reported similarly that MBOAT1/2 was a potent ferroptosis inhibitor in human osteosarcoma, which was independent of GPX4. Furthermore, members from the MBOAT family, regulated by sex hormone receptors, can confer benefits from hormone interference therapies in breast and prostate cancers. Nevertheless, given the distinct tumor origin and pathogenesis of MM compared to these cancers, there is limited theoretical basis for applying hormone-based interventions in MM treatment.

NR5A2 has been implicated in lipid metabolism through its role in regulating bile acid biosynthesis^15^ and intestinal epithelial glucocorticoid production^40^. But there is still no such report on its connection to lipid metabolism in MM. This study utilized lipidomic mass spectrometry analysis to explore the lipid regulatory function of NR5A2 in MM. As a result, overexpression of NR5A2 led to significantly reduced PE containing AA or AdrA, as well as ether lipids enriched with PUFA, highlighting the critical role of NR5A2 in reprogramming lipid metabolism in MM. Interestingly, these specific metabolites are recognized substrates for ferroptosis^41^,^42^, and their depletion is commonly associated with suppressed tumor cell ferroptosis. This further strengthens our hypothesis that NR5A2 can regulate ferroptosis via a non-GPX4-dependent pathway, providing new evidence for its involvement in tumor cell survival mechanisms.

Clinically, NR5A2 is often recognized as a marker of drug resistance in cancer therapy. Previous study confirmed that NR5A2 has a synergistic effect with NCOA3 in breast cancer, inhibiting ferroptosis by up-regulating NRF2 and leading to resistance to BET inhibitors^43^. In pancreatic cancer, NR5A2 mediates chemoresistance via the SOX2/MYC axis^44^. Additionally, evidence has shown that NR5A2 interacts with the glucocorticoid receptor in T-cell acute lymphoblastic leukemia, contributing to glucocorticoid resistance^16^. Similarly, in our study, NR5A2 overexpression in HMCLs led to significant Dexa resistance compared to the controls. Dexa serves as the cornerstone treatment for MM, its efficacy is a critical determinant of patient prognosis. Recent studies^9^,^45^ suggest that introducing appropriate metabolic substrates during cancer therapy can effectively reverse resistance and enhance therapeutic sensitivity. Based on our lipidomics analysis, we combined MA, OA, and AA with Dexa to treat HMCLs. Interestingly, only AA demonstrated a dose-dependent reversal of Dexa resistance. Our data indicate that AA reverses dexamethasone resistance in multiple myeloma cells by promoting ferroptosis. In conclusion, co-treatment with AA offers a promising approach to overcome Dexa resistance in MM, thereby profoundly enhancing the therapeutic efficacy for MM.

In this study, we elucidated the role of NR5A2 in promoting 1q+ MM cell proliferation and invasion and identified for the first time that the NR5A2-MBOAT1/MBOAT2 axis inhibits ferroptosis in MM cells. Overexpression of NR5A2 resulted in a marked reduction of ferroptosis-related substrates and induced resistance to Dexa. Notably, addition of AA reversed Dexa resistance in MM. These findings highlight a novel mechanism of drug resistance in MM and suggest potential therapeutic strategies targeting lipid metabolism to enhance treatment efficacy.

## Supplementary Material

Supplementary methods, figures and tables.
