# Supplementary material for: NR5A2 (located on chromosome 1q32) inhibits ferroptosis and promotes drug resistance by regulating phospholipid remodeling in multiple myeloma
Source: Int J Biol Sci. 2025 Sep 8;21(13):5789–801. doi: 10.7150/ijbs.113115 (PMC12509903; doi:10.7150/ijbs.113115)
Supplement: Supplementary file 1 — Supplementary methods, figures and tables. [file ijbsv21p5789s1.pdf]

Supplementary matrials

NR5A2 inhibits ferroptosis by regulating phospholipid remodeling in multiple myeloma

Supplementary Methods........ 2
Supplementary Figure 1........ 12
Supplementary Figure 2........ 13
Supplementary Figure 3........ 14
Supplementary Figure 4........ 15
Supplementary Figure 5........ 16
Supplementary Figure 6........ 17
Supplementary Figure 7........ 18
Supplementary Figure 8........ 19
Supplementary Figure 9........ 20
Supplementary Figure 10........ 21
Supplementary Figure 11........ 22
Supplementary Table 1........ 23
Supplementary Table 2........ 23
Supplementary Table 3........ 24
Supplementary Table 4........ 24
Supplementary Table 5........ 25

Supplementary Methods

Niacin skin test

Niacin-induced flushing responses were tested as described as previously(1). The platform comprised niacin methyl acetate solutions at six different concentrations (60mM, 20mM, 6.67mM, 2.23mM, 0.74mM, 0.25mM), liquid carrier slides with six holes, an image acquisition system, and image recognition software. Each of the six concentrations of niacin methyl acetate solution (0.05ml) was separately dispensed into the corresponding six holes of custom-made liquid carrier slides. The slides were then applied to the inner side of the subject's forearm and removed after 1 minute. The subject's arm was positioned within the image acquisition device, with the system automatically capturing one photo every 10 seconds for a total duration of 10 minutes, resulting in 62 photos per arm. Using the first photo as the reference baseline, professional image recognition software was employed to analyze and calculate the area of skin flushing reaction at each concentration, obtaining the total area of niacin-induced skin flushing reaction within 10 minutes for each subject.

Cell culture

Four human MM cell lines (HMCLs) were selected in this study: NCI-H929, AMO1, U266 and RPMI-8226. Cells were cultured in RPMI-1640 medium (HyClone, SH30809.01) supplemented with 10% fetal bovine serum (FBS) (Thermo Fisher Scientific, 10099141) in a humidified atmosphere containing 5% CO2/95% air at 37°C (Zhong Qiao Xin Zhou Biotechnology, Shanghai, China).

Construction of transfected stable and transient strain

The over-expression (OE) (Ubi-MCS-3FLAG-SV40-EGFP-IRES-puromycin) and knockdown (KD) (U6-MCS-Ubiquitin-Cherry-IRES-puromycin) lentivirus of NR5A2, were acquired from Genechem Co. Ltd (China). The OE and KD lentivirus vectors were infected into AMO1, RPMI-8226, NCI-H929 and U266 cells according to the manufacturer's protocols, respectively. The lentivirus-infected cell lines were subjected to selection with 1 μg/ml puromycin for 72 hours. MBOAT1 and MBOAT2 OE and KD plasmids were obtained from Genomeditec Co. Ltd (China). The vector

backbone of OE plasmids was 'CMV enhancer-MCS-3flag-polyA-EF1A-zsGreen-sv40-puromyci' while the vector backbone of KD plasmids was 'hU6-MCS-CBh-gcGFP-IRES-puromycin'.

Quantitative real-time polymerase chain reaction (qRT-PCR) and Western blot (WB)

RNA extraction and qRT-PCR, as well as protein extraction and Western blot (WB), were conducted following previously established protocols(2). Specific primer information is shown in attached **Table S4**, and specific antibody information is shown in attached **Table S5**.

Proteome profiling

Proteolytic Digestion, Peptide Labeling, and High-Performance Liquid Chromatography Separation

Based on the determined protein concentrations, 50 μg of protein from each sample was taken and diluted with lysis buffer to the same concentration and volume. Dithiothreitol (DTT) was added to the protein solution to a final concentration of 5 mM, mixed thoroughly, and incubated at 55°C for 30 minutes, followed by cooling on ice to room temperature. Subsequently, iodoacetamide was added to the solution to a final concentration of 10 mM, thoroughly mixed, and left in the dark at room temperature for 15 minutes. Acetone (6 times the volume) was then added, and the mixture was stored at -20°C for at least four hours, followed by centrifugation at 4°C, 8000 × g for 10 minutes to collect the precipitate. After evaporating the acetone for 2-3 minutes, the precipitate was dissolved in 100 μL of TEAB (200 mM), and 1 mg/ml trypsin-TPCK (1/50 of the sample mass) was added for overnight digestion at 37°C. The digested samples were freeze-dried, reconstituted with 100 mM TEAB buffer, and labeled in 1.5 mL Eppendorf tubes. TMT (iTRAQ) labeling reagents were equilibrated to room temperature, dissolved in anhydrous acetonitrile, added to the samples, mixed thoroughly, and left at room temperature for 1 hour. Finally, the reaction was terminated by adding 5% hydroxylamine, followed by freeze-drying and storage at -80°C.

The mixed samples were fractionated using a liquid chromatography system (Agilent

1100 HPLC) equipped with an Agilent Zorbax Extend-C18 narrow-bore column (2.1 × 150 mm, 5 μm). The gradient elution conditions were as follows: 0-8 min, 98% solvent A; 8-8.01 min, 98%-95% solvent A; 8.01-30 min, 95%-80% solvent A; 30-43 min, 80%-65% solvent A; 43-53 min, 65%-55% solvent A; 53-53.01 min, 55%-10% solvent A; 53.01-63 min, 10% solvent A; 63-63.01 min, 10%-98% solvent A; 63.01-68 min, 98% solvent A. Samples collected from 8 to 54 minutes were sequentially collected into centrifuge tubes numbered 1-15 every minute, with repeated collection from tube 1 to tube 15. After collection, the samples were dried and stored frozen until further mass spectrometric analysis. A total of 15 fractions were collected in this pre-fractionation process.

Liquid Chromatography-Mass Spectrometry (LC-MS/MS) Analysis

Each fraction was injected into the EASY-nLC 1200 liquid chromatography system (Thermo Fisher) at a flow rate of 300 nL/min for separation. The mobile phase consisted of solvent A (ACN-H2O-FA, 99.9:0.1, v/v) and solvent B (ACN-H2O-FA, 80:19.9:0.1, v/v/v). The gradient elution conditions were as follows: 0-50 min, 2-28% B; 50-60 min, 28-42% B; 60-65 min, 42-90% B; 65-75 min, 90% B. Peptides separated by the ultra-high-performance liquid chromatography system were introduced into the Q Exactive HF mass spectrometer (Thermo Fisher) for analysis. The mass spectrometric conditions were set as follows: The resolution of the first-level MS was set to 60,000, with an automatic gain control value of 3e6 and a maximum injection time of 50 ms. The mass spectrometry scan range was set from m/z 350 to 1500 for full scans, and the top 20 peaks were subjected to MS/MS scans. All MS/MS spectra were acquired using data-dependent acquisition in positive ion mode with higher-energy collisional dissociation (HCD) at a collision energy of 32. The resolution of MS/MS was set to 45,000, with an automatic gain control of 2e5 and a maximum injection time of 80 ms. The dynamic exclusion time was set to 30 s.

Qualitative and Quantitative Analysis of Proteins and Functional Analysis

The raw mass spectrometry data were imported into Proteome Discoverer software (Version 2.4, Thermo Fisher Scientific, USA) for spectrum analysis. The mass spectrometry search parameters included a parent ion tolerance of 10 ppm, a fragment

ion tolerance of 0.02 Da, fixed modifications of TMT (N-term, K) and Carbamidomethyl (C), variable modifications of Oxidation (M), and Acetyl (N-term), with a maximum missed cleavage site set to 2.

Comprehensive metabolomics-lipidomics

Sample Preparation

Cells were transferred into 2 mL centrifuge tubes with 600 μL of methanol-water (V: V=1:1, containing a mixture of internal standards at 4 μg/mL). 600 μL of chloroform was added, and the mixture was sonicated in an ice bath at 500 W for 3 minutes with a 6-second on and 4-second off cycle. The extraction was continued with sonication in an ice-water bath for 10 minutes. After standing at 4℃ for 30 minutes, the tubes were centrifuged for 10 minutes (12000 rpm, 4℃), and 400 μL of the lower layer was transferred to LC-MS sample vials and dried. 600 μL of chloroform-methanol (V: V=2:1) was added to the remaining centrifuge tubes, followed by vortexing for 30 seconds and sonication in an ice-water bath for 10 minutes. After standing at 4℃ for 30 minutes, the tubes were centrifuged for 10 minutes (12000 rpm, 4℃), and 400 μL of the lower layer was transferred back to the original LC-MS sample vials and dried. The lipid residue in the LC-MS vials was reconstituted with 300 μL of isopropanol-methanol (V: V=1:1), vortexed for 30 seconds, and sonicated in an ice-water bath for 3 minutes. The solution was then transferred to 1.5 mL EP tubes. After centrifugation for 10 minutes (12000 rpm, 4℃), 200 μL of the supernatant was transferred to LC-MS sample vials lined with inner tubes for injection into the LC-MS system. Quality control (QC) samples were prepared by mixing equal volumes of extraction from all samples, with the volume of each QC sample matching that of the individual samples.

LC-MS/MS Analysis

Metabolomic data analysis was performed by Shanghai Luming Biological Technology Co., Ltd (Shanghai, China). An ACQUITY UPLC I-Class Plus (Waters Corporation, Milford, USA) fitted with a Q-Exactive mass spectrometer equipped with a heated electrospray ionization (ESI) source (Thermo Fisher Scientific, Waltham, MA, USA) was used to analyze the metabolic profiling in both ESI positive and ESI negative ion modes. An ACQUITY UPLC HSS T3 column (1.8 μm, 2.1 ×

100 mm) was employed in both positive and negative modes. The binary gradient elution system consisted of (A) acetonitrile:water (60:40, v:v, containing 10mmol/L ammonium formate) and (B) acetonitrile:isopropanol (10:90, v:v, containing 10mmol/L ammonium formate), and separation was achieved using the following gradient: 0 min, 30% B; 3 min, 30% B; 5 min, 62% B; 15 min, 82% B; 16.5 min, 99% B; 18 min, 99% B; 18.1 min, 30% B; 22 min, 30% B. The flow rate was 0.35 mL/min and the column temperature was 45℃. All the samples were kept at 10℃ during the analysis. The injection volume was 5 μL. For positive mode: Heater Temp 300°C, Sheath Gas Flow rate 45 arb, Aux Gas Flow Rate 15 arb, Sweep Gas Flow Rate 1 arb, spray voltage 3.5 KV, Capillary Temp 320°C, S-F Level 50%. MS1 scan ranges: 120-1800. For negative mode: Heater Temp 300°C, Sheath Gas Flow rate 45 arb, ALens Rux Gas Flow Rate 15 arb, Sweep Gas Flow Rate 1 arb, spray voltage 3.1 KV, Capillary Temp 320°C, S-Lens RF Level 50%. MS1 scan ranges: 120-1800.

Data Preprocessing and Statistical Analysis

CCK-8 assay

Cell proliferation analysis was performed using a cell counting kit-8 (CCK-8) (Dojindo, Kumamoto, Japan) following the manufacturer's instructions. In brief, cells were seeded in 96-well plates and incubated for 0, 24, 48, and 72 hours. At each time point, 10 μl of sterile CCK-8 solution was added to each well and further incubated for 2 hours at 37°C. The absorbance at 450 nm was measured using a microplate reader.

Cell evasion assay

Trans-well migration assays were conducted to assess cell migration. After incubation with 200μL of FBS-free RPMI-1640 for 24 hours, 6×10^4 cells were added to the upper chambers of Matrigel Invasion Chambers (BD Pharmingen, Franklin Lakes, NJ, USA). Subsequently, 500μL of RPMI-1640 with 10% FBS was added to the lower compartment. After 24, 48, or 72 hours, the number of migrated cells was enumerated following staining of the membrane with Wright-Giemsa.

Transmission electron microscopy (TEM)

Following fixation with 2.5% neutral glutaraldehyde for 2 hours, cells were washed

six times with 0.1M phosphate buffer. Subsequently, cells were treated with 1% osmic acid for 1 hour. After gradient dehydration, resin infiltration, embedding, curing, semi-thin section positioning, ultra-thin sectioning, and uranium-lead double staining, the morphology of mitochondria in cells was observed using an electron microscope (Japan Electron Optics Laboratory Co., Ltd., JEM-1400 PLUS).

Measuring adenosine triphosphate (ATP) levels

HMCLs were homogenized with 150 μL of cold lysis buffer on ice and then centrifuged for 10 minutes at 12000 g at 4°C. After preparing the standard curve, 100 μL of ATP detection solution was added to a 96-well plate and incubated at room temperature for 5 minutes. Subsequently, 20 μL of cell supernatant was swiftly transferred into the 96-well plate and mixed. After 2 seconds, ATP detection was performed using a luminometer.

Quantifying intracellular reactive oxygen species (ROS)

Experimental cells were washed twice with PBS and then stained with 10μM DCFH-DA for 30 minutes. Fluorescence signals were assessed using a fluorescence microscope and Image J software was used for data processing.

Mitochondrial oxygen consumption rates (OCRs)

Briefly, cells were seeded at the optimal density of 6×10^5 cells/ml into Seahorse XFe96 cell culture microplates coated with poly-lysine (PDL) the night before the assay. For OCRs analysis, oligomycin, FCCP, and a combination of rotenone and antimycin A were sequentially injected into each well at specified times. Subsequently, data were analyzed using Seahorse XFe96 Wave software. Experiments were performed in triplicate and repeated independently three times.

In vivo assays

To investigate the impact of NR5A2 on multiple myeloma (MM) in vivo, we utilized a B-NDG (NOD.CB17-PrkdcscidIl2rgtm1/Bcgen, Beijing Biocytogen Co.) mouse model. All animals were housed under controlled conditions with a temperature of 22 ± 1°C, relative humidity of 50 ± 1%, and a 12-hour light/dark cycle. All animal procedures, including euthanasia, adhered to the regulations and guidelines of Fudan University's institutional animal care and were conducted following AAALAC and

IACUC guidelines.

B-NDG mice were subcutaneously inoculated with HMCLs (3×10^6 cells per mouse) in the right groin. Following tumor formation, tumor burden was evaluated by measuring tumor weight and estimating tumor size using a standard caliper. Tumor volumes were calculated using the formula volume = long diameter × short diameter × short diameter × 0.5.

Fe^{2+} staining

Experimental cells were washed twice with PBS and then stained with 5μM FeRhoNox-1 for 30 minutes. Fluorescence signals were assessed using a fluorescence microscope and Image J software was used for data processing.

Lipid peroxidation (MDA)

Lipid peroxidation was performed using a Lipid peroxidation Assay Kit (Abcam, ab118970)(3) In short, cells (2 × 106/well) were seeded into a 12-well plate and incubated at 37°C in 10% CO2. Then cells were homogenized in lysis solution on ice using a homogenizer and centrifuged at 13,000 g for 10 min. The supernatant was collected, mixed with TBA reagent, incubated at 95℃ for 60 min, and subjected to an optical density measurement at 532 nm on a microplate reader.

Oil red O stain

Detection of intracellular lipid levels using a commercial assay kit (Solarbio, G1262)(4). Cells were wash twice with PBS and then were fixed with Oil Red O fixative for 20-30 minutes. Discarded the fixative, rinsed twice with distilled water, and immerse in 60% isopropanol for 20-30 seconds. After removing the 60% isopropanol, added freshly prepared Oil Red O staining solution and incubated for 10-20 minutes. Washed 2-5 times with water until no excess staining solution remains and then added Mayer's hematoxylin staining solution and counterstain the nucleus for 1-2 minutes. After discarding the solution, rinsed 2-5 times with water, incubated with Oil Red O buffer for 1 minute, then discarded. Finally cells were covered with distilled water and observed under a microscope for photography and recording.

CUT-Tag

According to the instructions provided with the kit, the following steps were

sequentially performed(5): Buffer preparation, ConA Beads Pro treatment, cell collection, incubation of cell nuclei with ConA Beads Pro, primary antibody incubation, secondary antibody incubation, pA/G-Tnp Pro incubation, fragmentation, DNA Extract Beads Pro treatment, DNA extraction, DNA elution, and qPCR quantification.

Statistical analysis

Differentially expressed proteins were required to meet the criteria of $P < 0.05$ and fold change (FC) > 1.2 or FC $< 1/1.2$. Proteins were considered significantly upregulated when $P < 0.05$ and FC > 1.2, and significantly downregulated when $P < 0.05$ and FC $< 1/1.2$. The Gene Ontology (GO) database was used to analyze the biological processes (BP), cellular components (CC), and molecular functions (MF) of differentially expressed proteins based on their biological functions and classifications. The Kyoto Encyclopedia of Genes and Genomes (KEGG) database was utilized to analyze the main pathways involved by differentially expressed proteins. Protein-protein interaction (PPI) analysis of differentially expressed proteins was conducted using the STRING database, and a network of protein-protein interactions among differentially expressed proteins was constructed. The original Q Exactive LC-MS/MS data in raw format were processed by LipidSearch software for MSn and the exact mass-to-charge ratio (m/z) of parent ions. The molecular structure of lipids and the additive mode of its positive and negative ions were identified according to the parent ions and multi-stage mass spectrometry data of each individual sample. The results were aligned within a certain retention time range and combined into a single report to sort out the original data matrix. In each sample, all peak signals were normalized (i.e., the signal intensity of each peak was converted to the relative intensity in the spectrum, and then multiplied by 10000). The extracted data were then further processed by removing any peaks with a missing value (ion intensity = 0) in more than 50% of groups and by replacing the zero value by half of the minimum value. A data matrix was combined from the positive and negative ion data. The matrix was imported into R to carry out the Principle Component Analysis (PCA) to observe the overall distribution among the samples and the stability of the

whole analysis process. Orthogonal Partial Least-Squares-Discriminant Analysis (OPLS-DA) and Partial Least-Squares-Discriminant Analysis (PLS-DA) were utilized to distinguish the metabolites that differ between groups. To prevent overfitting, 7-fold cross-validation and 200 Response Permutation Testing (RPT) were used to evaluate the quality of the model. Variable Importance of Projection (VIP) values obtained from the OPLS-DA model were used to rank the overall contribution of each variable to group discrimination. A two-tailed Student's T-test was further used to verify whether the differences in metabolites between groups were significant. Differential metabolites were selected with VIP values greater than 1.0 and p-values less than 0.05.

Continuous variables were analyzed using Student's t-test, with all statistical tests being two-sided. Prognostic analysis was conducted using R software, version 3.6.0 (R Core Team, R Foundation for Statistical Computing). Kaplan-Meier (KM) survival curves were plotted to compare prognostic outcomes among different subgroups. For all figures: $p > 0.05$=ns; $p < 0.05$ = *; $p<0.01$ = **; $p<0.001$ = ***; $p<0.0001$ = ****.

References

1. Wang DD, Hu XW, Jiang J, Sun LY, Qing Y, Yang XH, et al. Attenuated and delayed niacin skin flushing in schizophrenia and affective disorders: A potential clinical auxiliary diagnostic marker. Schizophr Res. 2021 Apr;230:53–60.

2. Liu P, Xu B, Shen W, Zhu H, Wu W, Fu Y, et al. Dysregulation of TNFα-induced necroptotic signaling in chronic lymphocytic leukemia: suppression of CYLD gene by LEF1. Leukemia. 2012 Jun;26(6):1293–300.

3. Chen X, Song X, Li J, Zhang R, Yu C, Zhou Z, et al. Identification of HPCAL1 as a specific autophagy receptor involved in ferroptosis. Autophagy. 2023 Jan;19(1):54–74.

4. Gu C, Li P, Liu W, Zhou Y, Tan WS. The role of insulin in transdifferentiated hepatocyte proliferation and function in serum-free medium. J Cell Mol Med. 2019 Jun;23(6):4165–78.

5. Xie XD, Tang M, Yi SL, He Y, Chen SY, Zhao Y, et al. Polysaccharide of Asparagus cochinchinensis (Lour.) Merr regulates macrophage immune response

and epigenetic memory through TLR4-JNK/p38/ERK signaling pathway and histone modification. Phytomedicine. 2024 Feb;124:155294.

Supplementary Figures

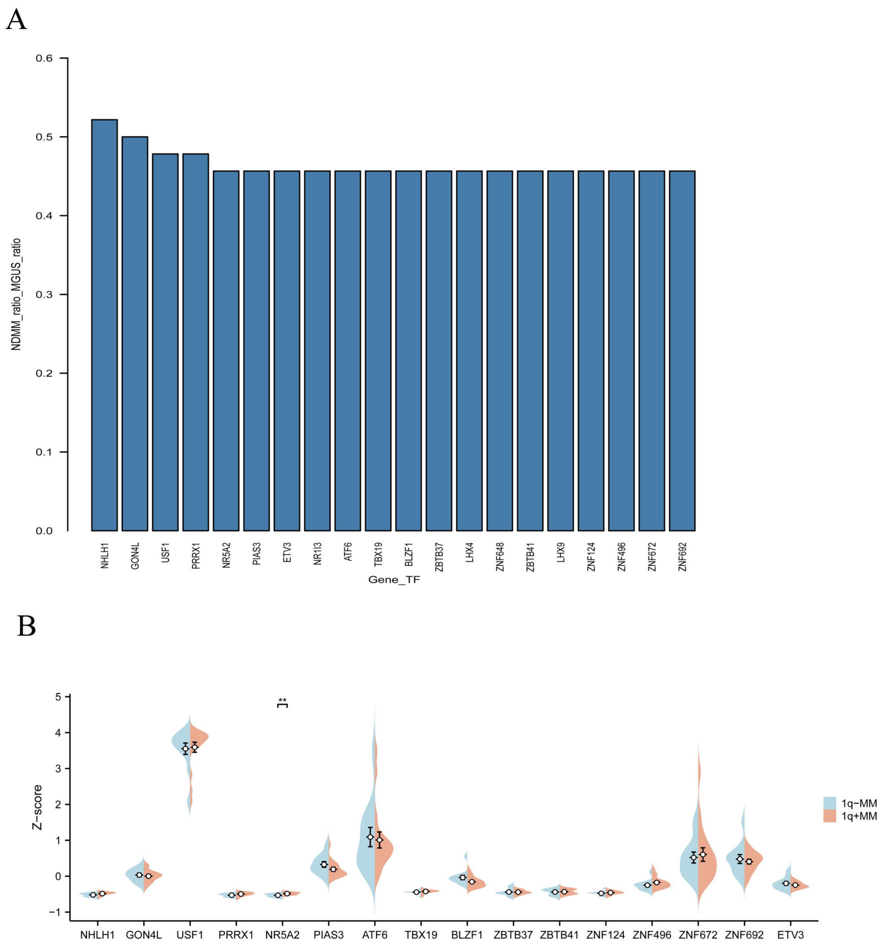

Supplement Figure 1

A. Determining the impact of each transcription factor on the PFS and OS of MM patients in the MMRF database (NHLH1， GON4L， NR5A2, USF1， PRRX1， PIAS3， ETV3， NR1I3， ATF6，TBX19，BLZF1，ZBTB37，LHX4，ZNF648，ZBTB41，LHX9，ZNF124，ZNF496，ZNF672 and ZNF692).

B. mRNA levels of these transcription factors were not significantly different between 1q+ and 1Q- MM patients.

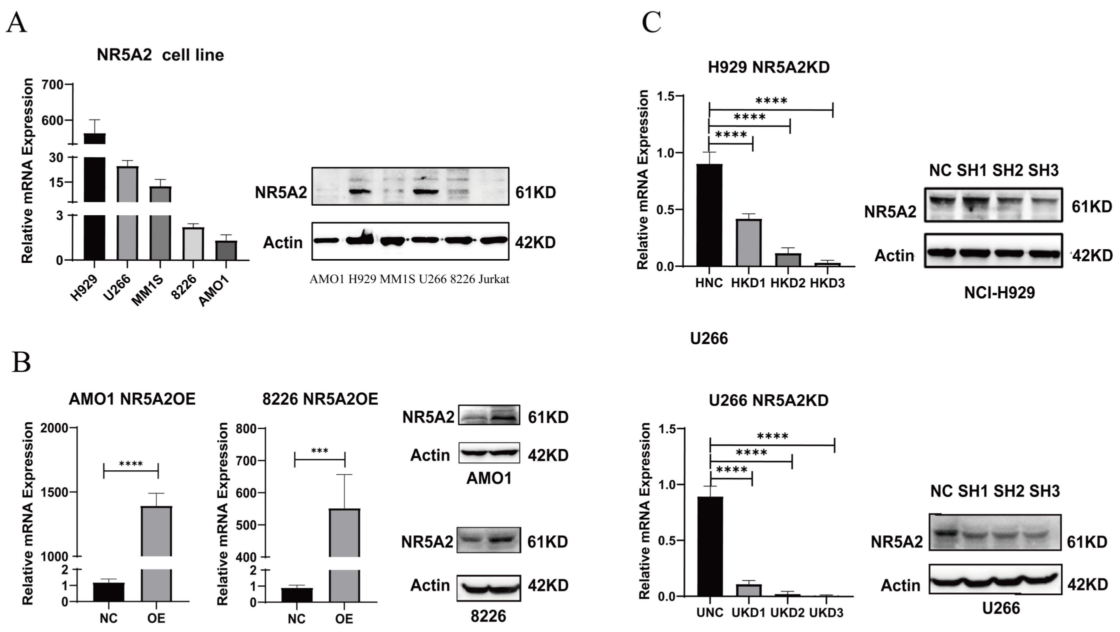

Supplement Figure 2

A. Validating the mRNA expression and protein levels of NR5A2 in HMCLs in our laboratory.

B. Verification of mRNA and protein levels of NR5A2 overexpression stable clones in AMO1-NR5A2-OE and RPMI-8226-NR5A2-OE cell lines.

C. Verification of mRNA and protein levels of NR5A2 knockdown stable clones in NCI-H929-NR5A2-KD and U266-NR5A2-KD cell lines.

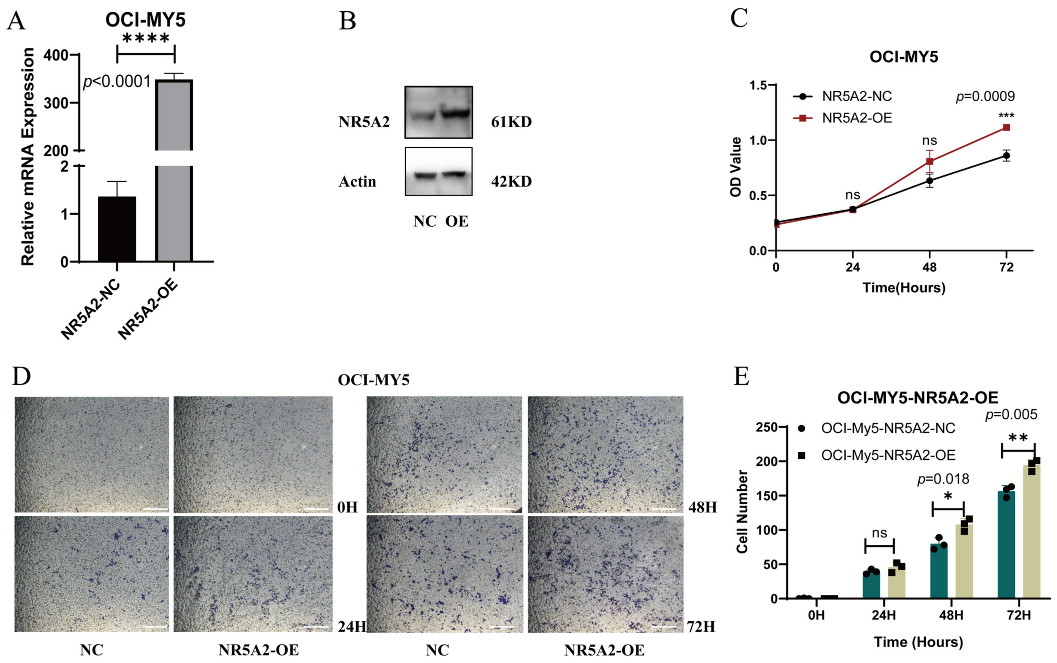

Supplement Figure3 NR5A2 in OCI-MY5 (1q- MM cells).

A. Overexpression of NR5A2 in OCI-MY5 verified in mRNA level.

B. verified in protein level.

C. OCI-MY5-NR5A2-OE had a quicker proliferation compared with the control group only at 72 hours (p=0.0009).

D. OCI-MY5-NR5A2-OE had higher invasion compared with the control group only at 48 (p=0.018) and 72 hours (p=0.005).

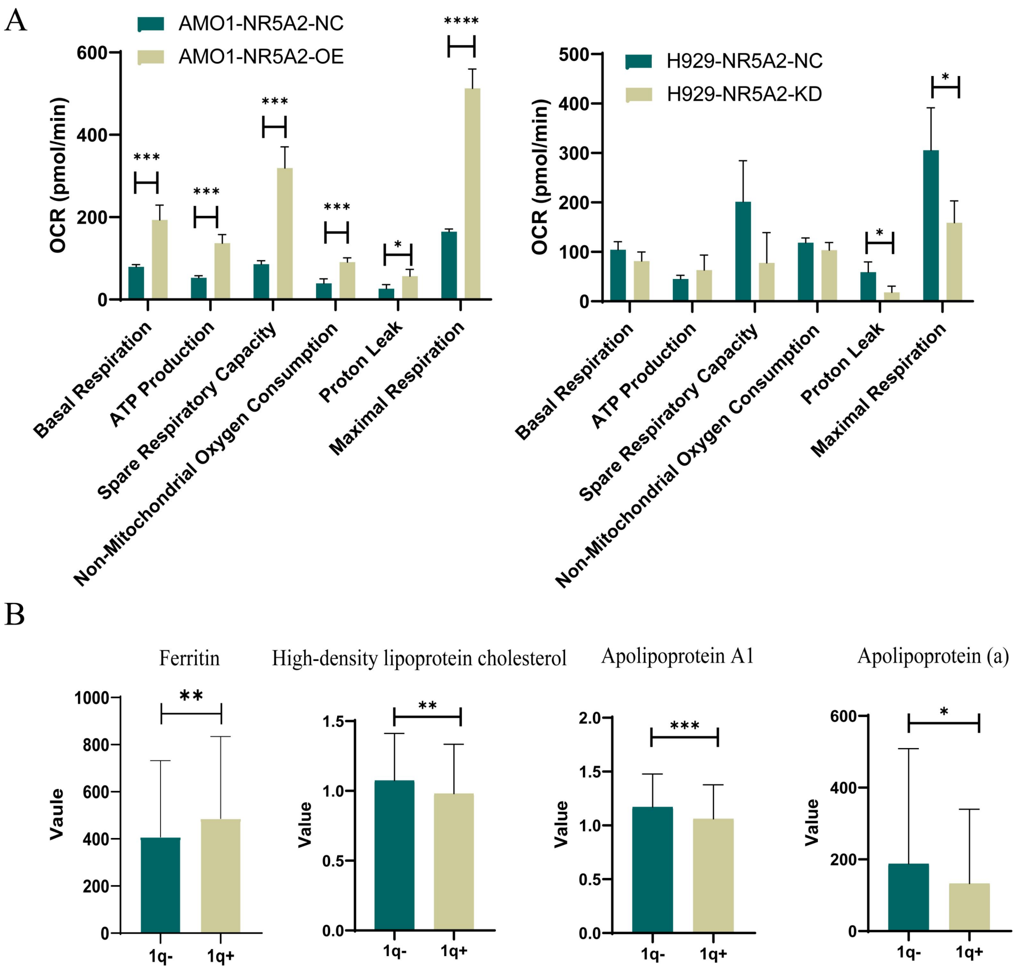

Supplement Figure 4

A. Changes in OCR-related parameters after NR5A2 OE/ KD.

B. Patients with 1q+ show elevated ferritin levels compared to those with 1q-, while high-density lipoprotein cholesterol, apolipoprotein A1, and lipoprotein (a) are reduced.

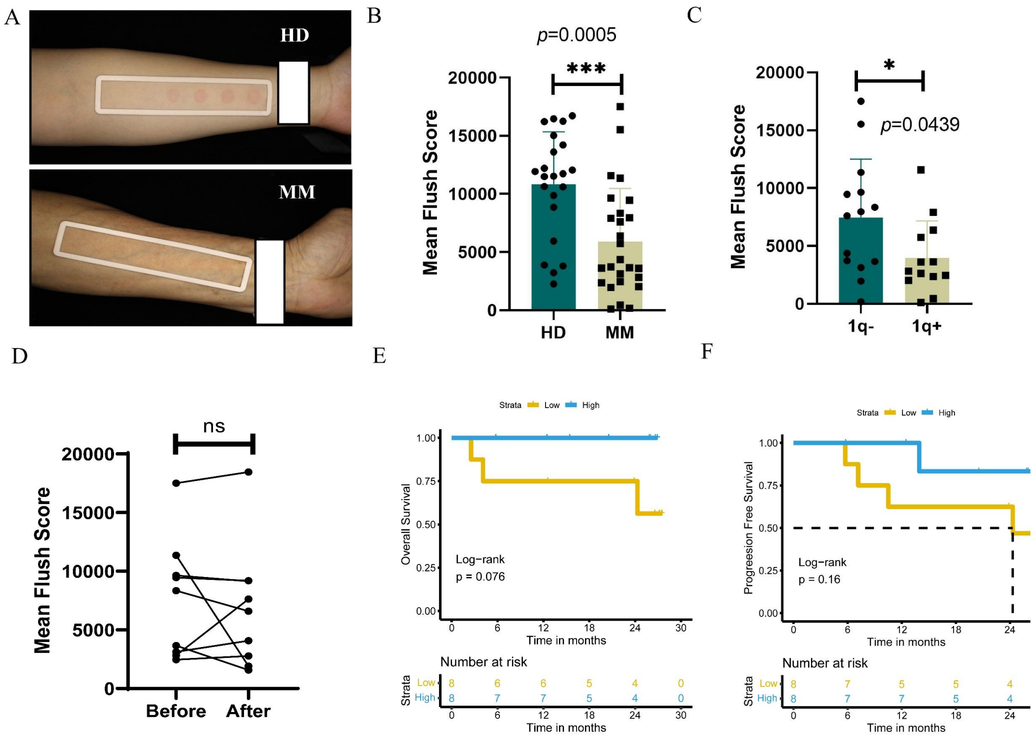

Supplement Figure 5 Niacin flushing in MM.

A. Diagram of niacin flushing reaction.

B. The niacin flushing response was significantly attenuated in MM versus healthy controls (p = 0.0005).

C. The niacin flushing response was significantly blunted in 1q+ MM patients compared with their 1q- counterparts (p = 0.0439).

D. Niacin flushing reactivity showed no significant alteration in MM patients pre- versus post-treatment.

E. F. A trend toward improved OS and PFS was observed in MM patients with high versus low niacin flushing reactivity.

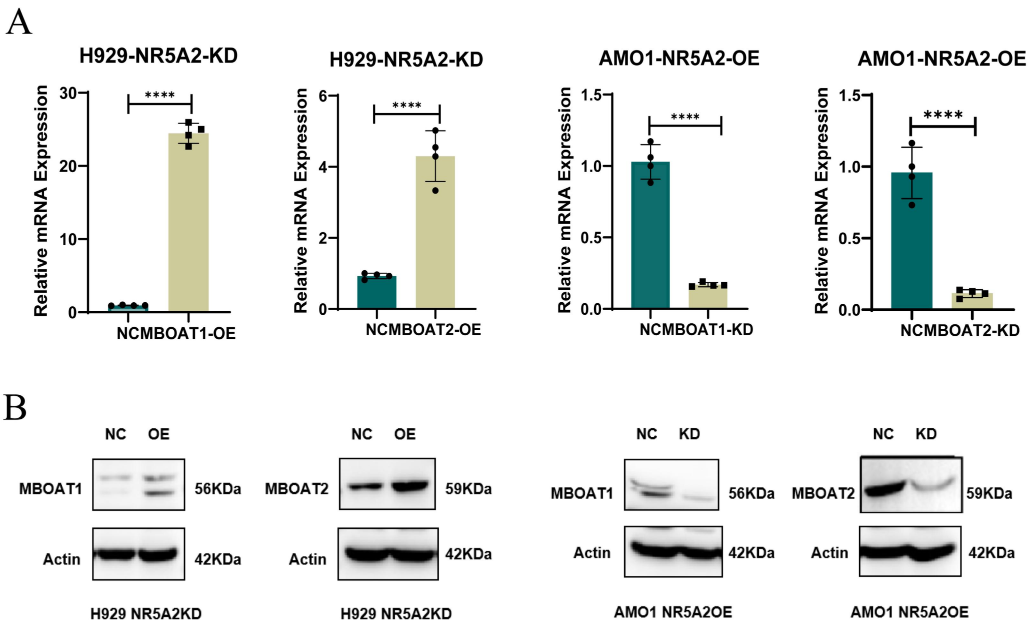

Supplement Figure 6

A. Validation of mRNA levels of MBOAT1 and MBOAT2 overexpression or knockdown in stable clones overexpressing or knocking down NR5A2.

B. Validation of protein levels of MBOAT1 and MBOAT2 overexpression or knockdown in stable clones overexpressing or knocking down NR5A2.

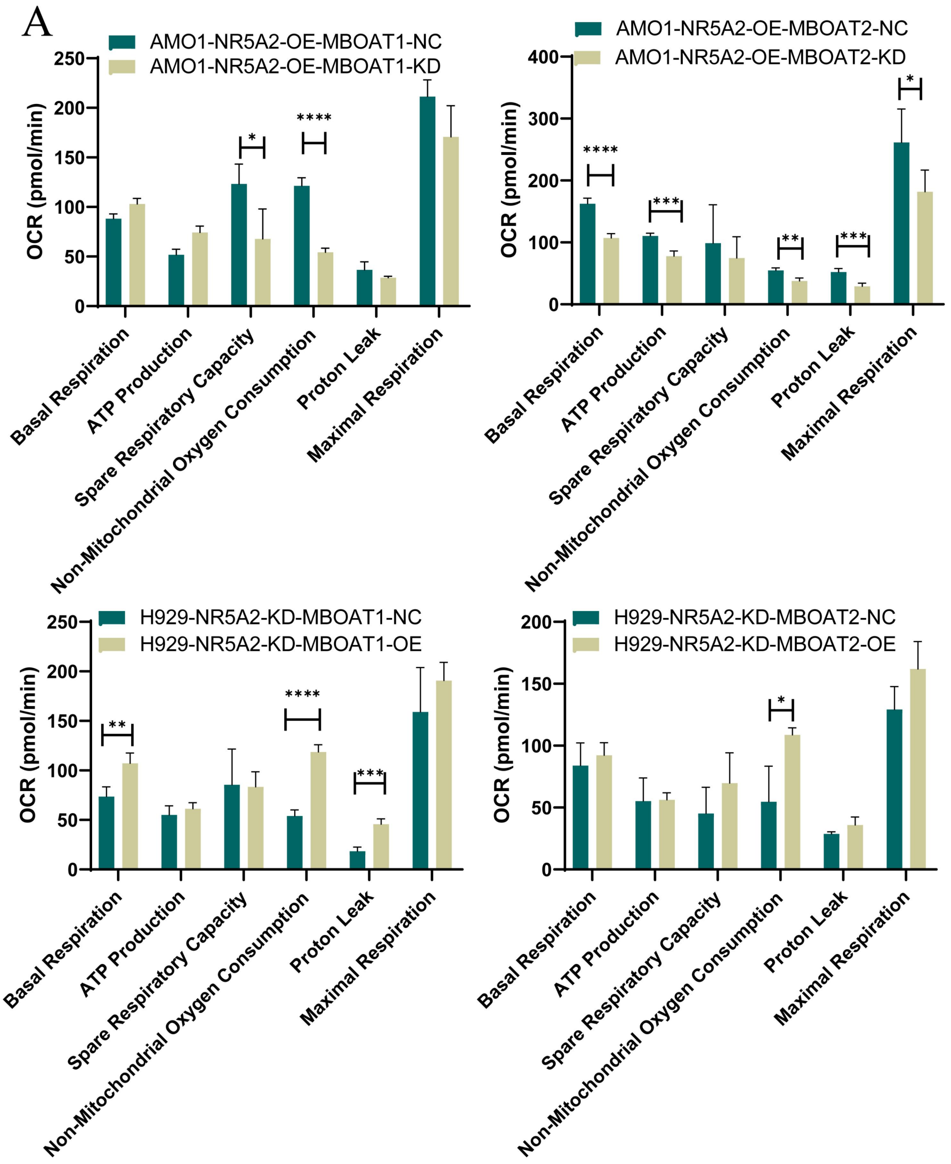

Supplement Figure 7 NR5A2 influenced mitochondrial energy metabolism-related indicators in HMCLs through MBOAT1 and MBOAT2.

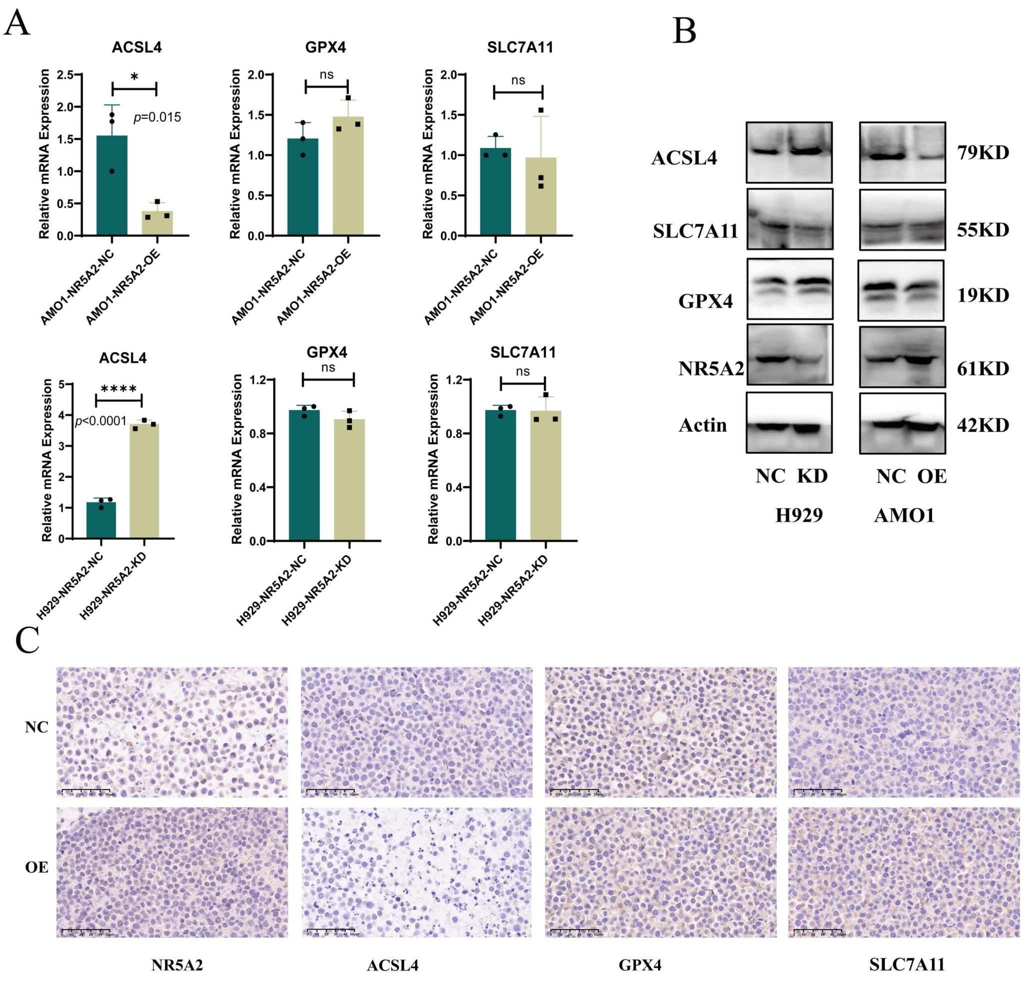

Supplement Figure 8 Mechanism underlying NR5A2-mediated ferroptosis suppression.

A. NR5A2 constrains ferroptosis in multiple myeloma through transcriptional repression of ACSL4 in mRNA level.

B. NR5A2 constrains ferroptosis in multiple myeloma through transcriptional repression of ACSL4 in protein level.

C. Immunohistochemical verification of xenograft tumor.

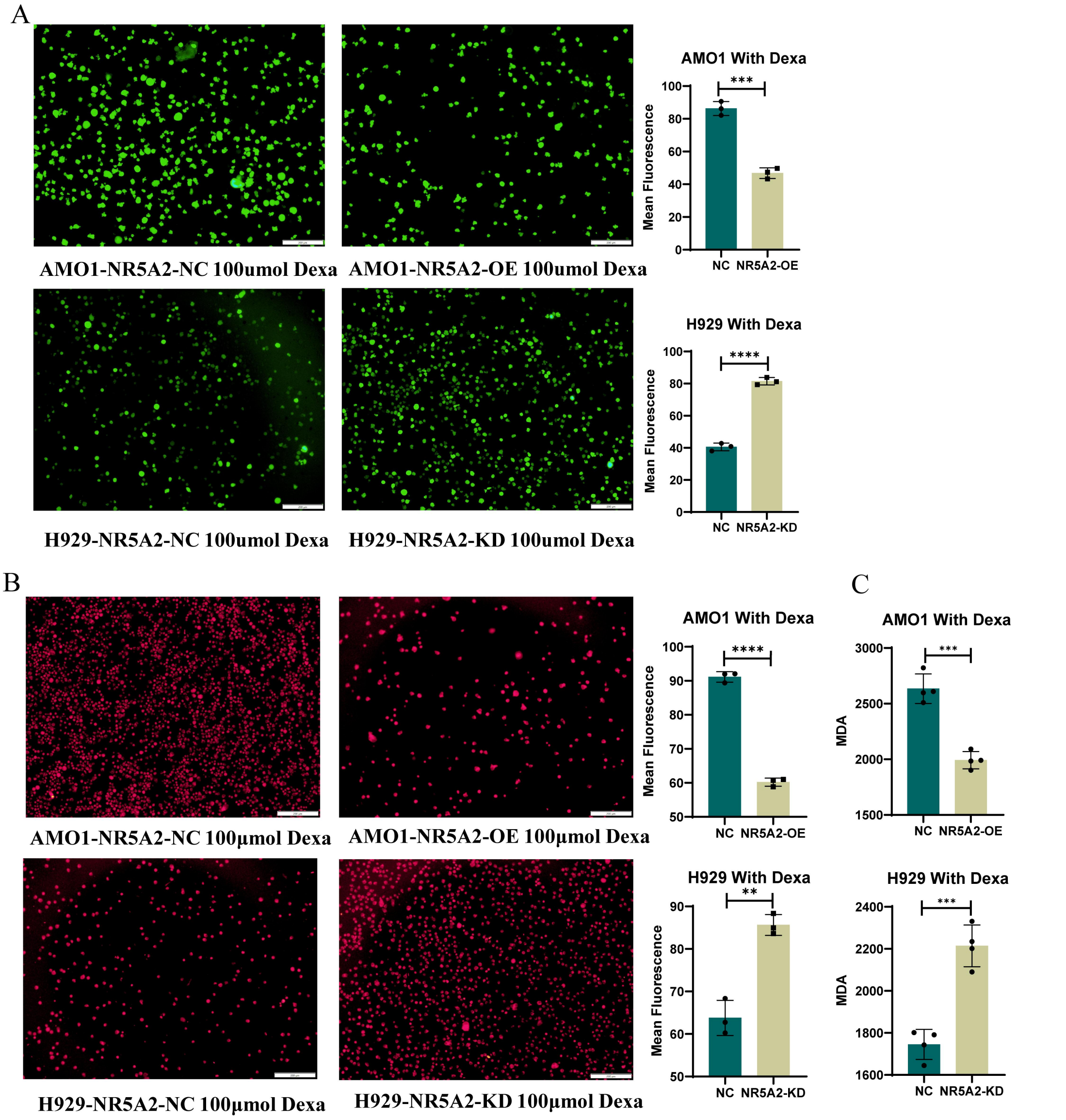

Supplement Figure 9

A. After treatment with 100μmol dexamethasone for 48 hours, the overexpression group of NR5A2 produced less ROS compared to the NC group, while the knockdown group of NR5A2 produced more ROS than the NC group.

B. After 48 hours of treatment with 100μmol dexamethasone, the overexpression group of NR5A2 showed fewer ROS compared to the NC group, while the knockdown group of NR5A2 showed more ROS compared to the NC group.

C. After 48 hours of treatment with 100μmol dexamethasone, the overexpression group of NR5A2 produced less MDA compared to the NC group, while the knockdown group of NR5A2 produced more MDA compared to the NC group.

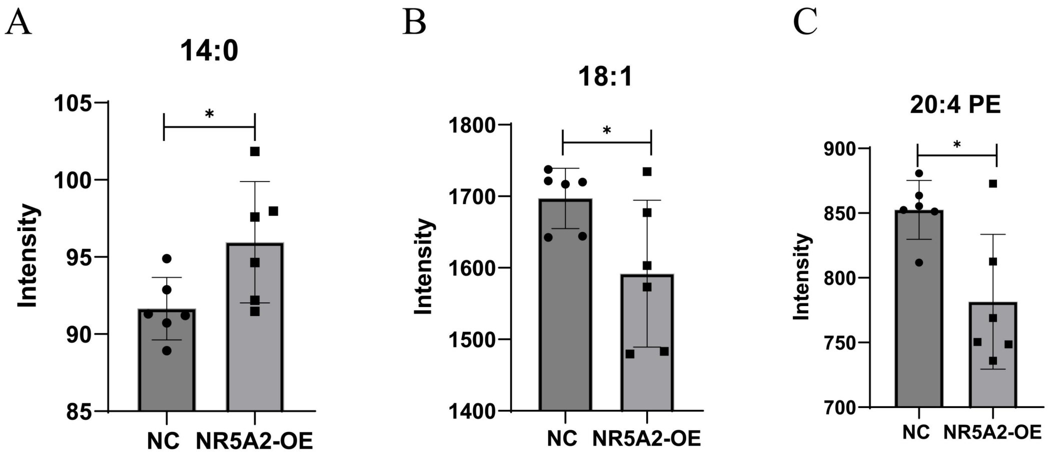

Supplement Figure 10

The expression of myristic acid (MA, 14:0), oleic acid (OA, 18:1), and arachidonic acid (AA, 20:4) between NR5A2-NC vs. NR5A2-OE.

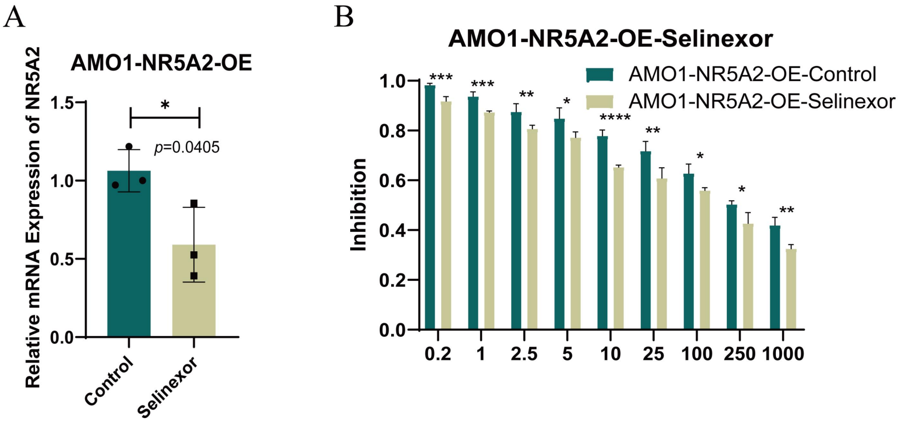

Supplement Figure 11

A. Selinexor inhibited NR5A2 transcription.

B. Selinexor antagonized NR5A2 overexpression-induced resistance of MM cells to dexamethasone.

Supplementary Tables

Table S1. Clinical Baseline Characteristics of 16 MM Patients and 15 Healthy Controls

	**HD**	**MM**
**Number**	22	27
**Gender, number (%)**		
Male	7 (31.8)	17(62.96)
Female	15 (68.2)	10(37.04)
**Age, mean, range**	42 (17.49)	66.07(10.93)
**Weight, mean (SD), kg**	62.23 (10.02)	62.89(13.43)
**Height, mean (SD), cm**	166.59 (5.42)	164.44(8.84)
**BMI, mean (SD), kg/m2**	22.39 (3.17)	23.07(3.45)

Abbreviations: HD, Healthy donors; MM, Multiple myeloma; SD, Standard Deviation; BMI, Body Mass Index.

Table S2. The Clinical Information of the Patient Cohort Undergoing Whole Exome Sequencing

**Clinical characteristics**	**Patients**
**Number**	76
**Age, Median (Range)**	63 (41-87)
**Male, Number (%)**	47 (61.8)
**ISS, Number (%)**	
I	22 (28.5)
II	17 (22.0)
III	36 (46.7)

Abbreviations: ISS, International Stage System.

Table S3. The Clinical Information of the Patient Cohort with Ferritin and FISH Test Results

Clinical characteristics	Patients
**Number**	584
**Age, Median (Range)**	65 (32-87)
**Male, Number (%)**	362 (61.9)
**ISS, Number (%)**	
I	168 (28.7)
II	159 (27.2)
III	248 (42.4)

Abbreviations: ISS, International Stage System.

Table S4. The primers for qRT-PCR were purchased from Shanghai Sangon Biotech Co., Ltd. (Shanghai Synthesis Department).

Gene	Sequence (5'-3')
NR5A2 forward	CTTTGTCCCGTGTGTGGAGAT
NR5A2 reverse	GTCGGCCCTTACAGCTTCTA
MBOAT1 forward	TGTGCTGGTGTTAATGTGCTAT
MBOAT1 reverse	GGCTGATGTGGCATATTGTAAGA
MBOAT2 forward	CTCGCTGGGACTTAATTTCCAA
MBOAT2 reverse	GGTTCGTTCATAACACACCCTT
GAPDH forward	GCCCAATACGACCAAATCC
GAPDH reverse	CACCACATCGCTCAGACAC
GPX4 forward	GAGGCAAGACCGAAGTAAACTAC
GPX4 reverse	CCGAACTGGTTACACGGGAA
MBOAT1-CUT&Tag - forward	CTCCAGCAGGAGTGAGTGTG

MBOAT1-CUT&Tag - reverse	CTTCCAAACTCGCAAGCCAC
MBOAT2-CUT&Tag - forward	GTAGGTTTGGACTGGCAGCA
MBOAT2-CUT&Tag - reverse	CGTAGCACCACGCATTACTC

Table S5. Antibody information

**Gene**	**Manufacturer and product No**	**species**
NR5A2	Affinity；DF8470q	Rabbit
MBOAT1	Fisher Scientific；PIPA5-43193	Rabbit
MBOAT2	Novus Biologicals；NBP182236	Rabbit
GPX4	Affinity; DF6701	Rabbit
GAPDH	Beyotime；AF0006	Mouse
Actin	Proteintech；60004-1	Mouse
NR5A2-CUT&Tag	Santa cruz；sc-393369	Mouse
IgG-CUT&Tag	Cell Signaling Technology; 2729	Rabbit
